# A Biophysical Study with Carbohydrate Derivatives Explains the Molecular Basis of Monosaccharide Selectivity of the *Pseudomonas aeruginosa* Lectin LecB

**DOI:** 10.1371/journal.pone.0112822

**Published:** 2014-11-21

**Authors:** Roman Sommer, Thomas E. Exner, Alexander Titz

**Affiliations:** 1 Helmholtz Institute for Pharmaceutical Research Saarland (HIPS), Campus C 2.3, D-66123, Saarbrücken, Germany; 2 Department of Chemistry and Graduate School Chemical Biology, University of Konstanz, D-78457, Konstanz, Germany; 3 Theoretical Medicinal Chemistry and Biophysics, Institute of Pharmacy, University of Tübingen, D-72076, Tübingen, Germany; The Ohio State University, United States of America

## Abstract

The rise of resistances against antibiotics in bacteria is a major threat for public health and demands the development of novel antibacterial therapies. Infections with *Pseudomonas aeruginosa* are a severe problem for hospitalized patients and for patients suffering from cystic fibrosis. These bacteria can form biofilms and thereby increase their resistance towards antibiotics. The bacterial lectin LecB was shown to be necessary for biofilm formation and the inhibition with its carbohydrate ligands resulted in reduced amounts of biofilm. The natural ligands for LecB are glycosides of d-mannose and l-fucose, the latter displaying an unusual strong affinity. Interestingly, although mannosides are much weaker ligands for LecB, they do form an additional hydrogen bond with the protein in the crystal structure. To analyze the individual contributions of the methyl group in fucosides and the hydroxymethyl group in mannosides to the binding, we designed and synthesized derivatives of these saccharides. We report glycomimetic inhibitors that dissect the individual interactions of their saccharide precursors with LecB and give insight into the biophysics of binding by LecB. Furthermore, theoretical calculations supported by experimental thermodynamic data suggest a perturbed hydrogen bonding network for mannose derivatives as molecular basis for the selectivity of LecB for fucosides. Knowledge gained on the mode of interaction of LecB with its ligands at ambient conditions will be useful for future drug design.

## Introduction

Infections with the Gram-negative, opportunistic pathogen *Pseudomonas aeruginosa* (*P. aeruginosa*) are a severe problem for hospitalized and immuno-compromised patients [1], [2]. In addition, the viscous mucus secreted by lung tissue of patients suffering from cystic fibrosis (CF) provides a good habitat for *P. aeruginosa* [3]. Infections are observed in up to 80% of CF patients, leading to chronic pneumonia and lung failure. This bacterium can form biofilms and thereby increases its resistance towards antibiotic treatment [4], [5]. The bacterial lectin LecB (also called PA-IIL), a virulence factor [6] of *P. aeruginosa*, is necessary for biofilm formation [7] and its inhibition with carbohydrate ligands results in reduced biofilm growth [8]. The structure of LecB in complex with its monosaccharide ligands l-fucose and d-mannose was determined by X-ray crystallography [9], [10].

Since the unusual strong interaction of l-fucose with LecB (K_d_ = 430 nM [11] for methyl α-l-fucoside, **1**) and the approximately 150-fold weaker interaction of d-mannose (K_d_ = 71 µM [11] for methyl α-d-mannoside, **2**) was reported first by Gilboa-Garber *et al*. [12], numerous fucosides were examined as LecB inhibitors due to the potency of this binding [13], [14]. Recently, we reported on terminally modified mannosides as potent LecB inhibitors with low micromolar binding affinities [15]. In order to further improve LecB inhibitors as therapeutics against chronic *P. aeruginosa* infections, we consider a detailed understanding of the contributions of the individual pharmacophores or functional groups in fucosides and mannosides to the overall binding affinity to be essential.

**Figure 1:**
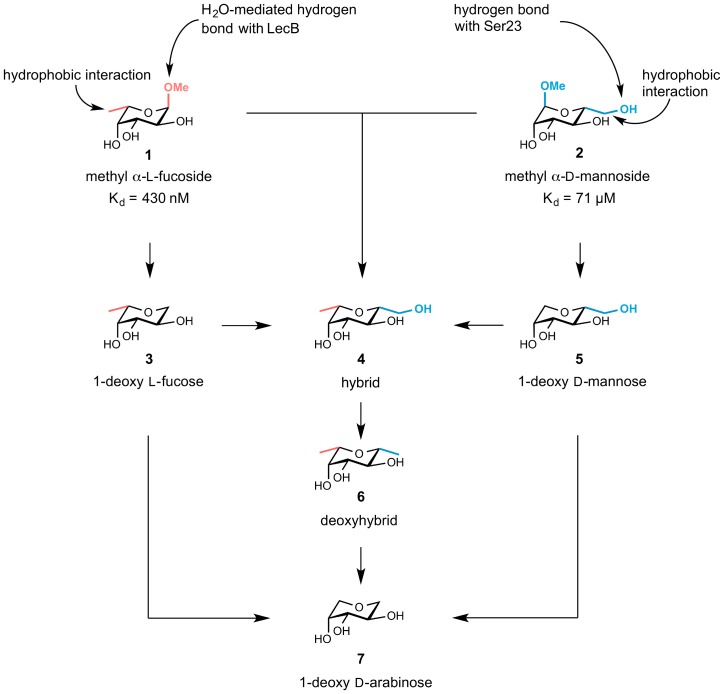
Illustration of the interactions of LecB with 1 and 2 based on the X-ray structures [9], [10], [44] of the carbohydrates with LecB. To elucidate the individual contributions of substituents adjacent to the ring oxygen, target derivatives 3–7 were designed. A combination of moieties forming attractive interactions with LecB in hybrid structure 4 may lead to synergistic effects for binding. Hydroxy groups necessary for Ca^2+^-binding are shown in black. Moieties derived from l-fucose are shown in red and from d-mannose in blue. Dissociation constants (K_d_) are taken from Sabin *et al.* [11].

The interaction of LecB with its natural ligands, fucosides (*e.g.*, **1**) and mannosides (*e.g.*, **2**) (Figure 1) is mediated by two Ca^2+^ ions in the binding site, which coordinate the ligand though their 2-, 3- and 4-hydroxy groups. Furthermore, an additional lipophilic contact of the equatorial methyl group in fucosides served as an explanation for their unusual high affinity [10]. Interestingly, although mannosides form an additional hydrogen bond *via* O-6 with Ser23 in the crystal structure [9], they are much weaker ligands for LecB in solution.

To elucidate the individual contributions of the lipophilic interaction of the C-6 methyl group in methyl α-l-fucoside (**1**) and the hydrogen-bond forming hydroxymethyl group in methyl α-d-mannoside (**2**) to binding with LecB, a distinct set of derivatives was synthesized and their interactions with LecB characterized. By using a competitive binding assay and thermodynamic techniques, the contribution of these substituents to the binding affinity was quantified. Theoretical analyses using molecular dynamics simulations and predictions of free energies of binding revealed a destabilization of a hydrogen-bonding network of Asp96 in LecB, resulting from steric hindrance in mannose-derived ligands, as molecular basis for the increased binding affinity of fucosides over mannosides.

## Experimental

### Chemical Syntheses

Nuclear magnetic resonance (NMR) spectroscopy was performed on a Bruker Avance III 400 UltraShield spectrometer at 400 MHz (^1^H) or 101 MHz (^13^C). Chemical shifts are given in ppm and were calibrated on residual solvent peaks as internal standard [16]. Multiplicities were specified as s (singlet), d (doublet), t (triplet) or m (multiplet). The signals were assigned with the help of ^1^H,^1^H-COSY, DEPT-135-edited ^1^H,^13^C-HSQC and ^1^H,^13^C-HMBC experiments. High resolution mass spectra were obtained on a Bruker micrOTOF II ESI spectrometer and the data were analyzed using DataAnalysis from Bruker. HPLC was performed on a Shimadzu HPLC system. Thin layer chromatography (TLC) was performed using silica gel 60 coated aluminum sheets containing fluorescence indicator (Merck KGaA, Darmstadt, Germany) using UV light (254 nm) and by charring either in anisaldehyde solution (1% v/v 4-methoxybenzaldehyde, 2% v/v concentrated H_2_SO_4_ in EtOH), in aqueous KMnO_4_ solution or in a molybdate solution (a 0.02 M solution of ammonium cerium sulfate dihydrate and ammonium molybdate tetrahydrate in aqueous 10% H2SO4) with heating. Medium pressure liquid chromatography (MPLC) was performed on a Teledyne Isco Combiflash Rf200 system using pre-packed silica gel 60 columns from Teledyne Isco, SiliCycle or Macherey-Nagel. Commercial chemicals and solvents were used without further purification. Methyl α-d-mannoside was purchased from Sigma Aldrich (Germany), l-fucose from Dextra Laboratories (Reading, UK), methyl β-d-arabinoside from TCI Europe, methyl α-l-fucoside and methyl β-l-fucoside from Carbosynth Ltd. (UK). Deuterated solvents were purchased from Eurisotop (Saarbrücken, Germany).

Compound **9** was prepared by following the procedures from Bordoni *et al*. [17] and Kondo *et al*. [18] 1-deoxy d-mannose (**5**) was synthesized as reported by Guo *et al*. [19] and β-l-fucopyranosyl nitromethane (**16**) was prepared as described by Phiasivongsa *et al*. [20] Compounds **20** and **21** were prepared by following the protocols from Nishi and Tanimoto [21] and Daniellou and Narvor [22].

#### 1-Deoxy l-fucose (3).

To a solution of methyl 2,3,4-tri-O-benzyl-l-fucoside (**9**) (1.82 g, 4.06 mmol) and triethylsilane (1.78 mL, 11.1 mmol) in CH_2_Cl_2_ (11.2 mL), trimethylsilyl trifluoromethanesulfonate (1.51 mL, 8.35 mmol) was added dropwise at 0°C and stirred for 15 min. The mixture was warmed to r.t. and stirred for additional 15 h. The reaction was quenched with saturated NaHCO_3_. The aqueous phase was extracted with CH_2_Cl_2_ (3×20 mL), the combined organic layers were dried over Na_2_SO_4_ and the solvent was removed under reduced pressure. The residue was purified by MPLC (PE to PE/EtOAc  = 8∶1) to give 1-deoxy-2,3,4-tri-O-benzyl-l-fucose (**10**) (1.12 g, 2.67 mmol, 66%). **10** was dissolved in MeOH (20 mL) and stirred under hydrogen atmosphere (1 bar) with 10% Pd-C (300 mg, 10 mol%) at r.t. for 16 h. The mixture was filtered through celite and the solvent was removed under reduced pressure. The residue was purified by MPLC (CH_2_Cl_2_ to CH_2_Cl_2_/EtOH  = 6∶1) to give 1-deoxy l-fucose (**3**) (315 mg, 2.13 mmol, 80%) as colorless solid. The synthesis of compound **3** was first reported by Carpintero *et al.* using a different synthetic route [23]. ^1^H NMR (400 MHz, MeOH-d4) δ 3.87 (dd, J = 10.9, 5.5 Hz, 1H, CH_2_), 3.76 (ddd, J = 10.4, 9.47, 5.5 Hz, 1H, H-2), 3.65 (dd, J = 3.4, 1.1 Hz, 1H, H-4), 3.53 (qd, J = 6.5, 1.1 Hz, 1H, H-5), 3.39 (dd, J = 9.4, 3.4 Hz, 1H, H-3), 3.10 (t, J = 10.6 Hz, 1H, CH_2_), 1.24 (d, J = 6.5 Hz, 3H, CH_3_). ^13^C NMR (101 MHz, MeOH-d4) δ 76.69 (C-3), 76.48 (C-5), 73.46 (C-4), 71.17 (C-1), 68.17 (C-2), 17.11 (CH_3_). HR-MS calcd. for C_6_H_12_NaO_4_^+^: 171.0628; found: 171.0622.

#### 1-Deoxy D-arabinose (7).

For **7**, the same procedure as for 1-deoxy l-fucose (**3**) was used with methyl arabinoside **13** as starting material. After purification by MPLC (CH_2_Cl_2_ to CH_2_Cl_2_/EtOH  = 6∶1) **7** was isolated as colorless solid (57%; 3 steps). ^1^H NMR (400 MHz, MeOH-d4) δ 3.89–3.84 (m, 1H, CH), 3.82 (dd, *J* = 11.2, 4.1 Hz, 1H, CH_2_), 3.78–3.68 (m, 2H, CH, CH_2_), 3.56 (dd, *J* = 7.5, 3.4 Hz, 1H, CH), 3.50 (dd, *J* = 11.8, 2.6 Hz, 1H, CH_2_), 3.20 (dd, *J* = 11.2, 7.4 Hz, 1H, CH_2_); ^13^C NMR (101 MHz, MeOH-d4) δ 74.25, 70.49, 70.41, 69.37, 69.07. HR-MS calcd. for C_5_H_10_NaO_4_^+^: 157.0471; found: 157.0489.

#### β-L-fucopyranosyl methanol (4), named hybrid 4.

β-l-fucopyranosyl nitromethane (**16**) (351 mg, 1.69 mmol) and 5% Pt-C (131 mg, 10%) were suspended in 15 mL MeOH and 340 mL HCl (1 M) and stirred at r.t. for 2 d under H_2_-atmosphere. The suspension was filtered over celite and the solvent was removed under reduced pressure to give 328 mg of crude product β-l-fucopyranosyl methylamine **17** as hydrochloride. Amine **17** was dissolved in H_2_O (5.6 mL) and AcOH (357 µL) and NaNO_2_ (502 mg, 6.25 mmol) were added at 0°C and the reaction was allowed to warm to r.t.. After removing the solvent and purification by MPLC (EtOAc/EtOH  = 1∶1) β-l-fucopyranosyl methanol (**4**) (25 mg, 0.14 mmol, 25%) was obtained as colorless solid. ^1^H NMR (400 MHz, MeOH-d4) δ 3.86–3.80 (m, 1H, CH_2_), 3.70–3.57 (m, 3H, CH_2_, H-4, H-5), 3.52 (m, 1H, H-2), 3.44 (dd, J = 9.4, 3.2 Hz, 1H, H-3), 3.18 (m, 1H, H-1), 1.25 (d, J = 6.5 Hz, 3H, CH_3_). ^13^C NMR (101 MHz, MeOH-d4) δ 81.97 (C-1), 76.70 (C-3), 75.49 (C-5), 73.75 (C-6), 68.75 (C-2), 63.16 (CH_2_), 17.15 (CH_3_). HR-MS calcd. for C_7_H_14_NaO_5_^+^: 201.0733; found: 201.0728.

#### β-L-fucopyranosyl bis(ethylthio)methane (18).

β-l-fucopyranosyl nitromethane (**16**) (40 mg, 0.19 mmol) was added to a solution of NaOMe in MeOH (0.35 M, 2.2 mL) and stirred at r.t. for 30 min. The light yellow solution was added to a solution of AcCl in EtSH (0.5 M, 4.4 mL) at 0°C over 15 min and stirring was continued for 1.25 h and then an additional 1 h at r.t.. A mixture of both Amberlite H^+^ and OH^-^ was added, the reaction was stirred for 10 min and filtered. The solvent was removed under reduced pressure and the residue purified by MPLC (CH_2_Cl_2_ to CH_2_Cl_2_/EtOH  = 8∶1) to give β-l-fucopyranosyl bis(ethylthio)methane (**18**, 15 mg, 0.05 mmol, 28%) as colorless solid. ^1^H NMR (400 MHz, MeOH-d4) δ 4.21 (d, J = 1.8 Hz, 1H, CH(SCH_2_CH_3_)_2_), 3.90 (t, J = 9.3 Hz, 1H, H-2), 3.62 (dd, J = 3.6, 1.2 Hz, 1H, H-4), 3.55 (qd, J = 6.5, 1.2 Hz, 1H, H-5), 3.49 (dd, J = 9.3, 1.8 Hz, 1H, H-1), 3.44 (dd, J = 9.4, 3.5 Hz, 1H, H-3), 2.73 (m, 4H, CH(SCH_2_CH_3_)_2_), 1.30–1.17 (m, 9H, H-6, CH(SCH_2_CH_3_)_2_); ^13^C NMR (101 MHz, MeOH-d4) δ 86.26 (C-1), 76.58 (C-3), 76.00 (C-5), 73.54 (C-4), 69.86 (C-2), 53.20 (CH(SCH_2_CH_3_)_2_), 26.13 (CH(SCH_2_CH_3_)_2_), 26.05 (CH(SCH_2_CH_3_)_2_), 17.07 (C-6), 15.07 (CH(SCH_2_ CH_3_)_2_), 14.91 (CH(SCH_2_ CH_3_)_2_); HR-MS calcd. for C_11_H_22_NaO_4_S_2_^+^: 305.0852; found: 305.0819. Furthermore, a side product was isolated and the structure was assigned to **19** (14 mg, 0.05 mmol, 29%). ^1^H NMR (400 MHz, MeOH-d4) δ 4.06 (t, *J* = 9.4 Hz, 1H, H-2), 3.94 (d, *J* = 9.5 Hz, 1H, H-1), 3.73 – 3.63 (m, 2H, H-4, H-5), 3.51 (dd, *J* = 9.4, 3.3 Hz, 1H, H-3), 3.05 (qq, *J* = 12.4, 7.4 Hz, 2H, CH_2_), 1.29 (t, *J* = 7.4 Hz, 3H, CH_3_), 1.25 (d, *J* = 6.5 Hz, 3H, C-6); ^13^C NMR (101 MHz, MeOH-d4) δ 151.93 (CNOHSEt), 80.86 (C-1), 76.17, 76.02, 73.31 (C-3, C-4, C-5), 69.47 (C-2), 24.72 (CH_2_), 17.15 (C-6), 15.56 (CH_3_); HR-MS calcd. for C_9_H_18_NO_5_S^+^: 252.0906; found: 252.0908.

#### β-L-fucopyranosyl methane (6), named deoxyhybrid 6.

Dithioacetal **18** (15 mg, 0.05 mmol) was stirred in a suspension of EtOH (2 mL) and Raney Ni 2800 in water (2 mL) for 30 min at 80°C. The solids were removed by filtration over celite and the solvent was removed under reduced pressure. The residue was purified by MPLC (CH_2_Cl_2_ to CH_2_Cl_2_/EtOH  = 9∶1) to give the title compound **6** (8.0 mg, 0.05 mmol, 93%) as colorless solid. ^1^H NMR (600 MHz, MeOH-d4) δ 3.63 (dd, J = 3.5, 1.1 Hz, 1H, H-4), 3.57 (qd, J = 6.5, 1.1 Hz, 1H, H-5), 3.40 (dd, J = 9.4, 3.4 Hz, 1H, H-3), 3.28 (t, J = 9.3 Hz, 1H, H-2), 3.19 (dq, J = 9.2, 6.1 Hz, 1H, H-1), 1.25 (d, J = 6.1 Hz, 3H, CH_3_-1), 1.22 (d, J = 6.5 Hz, 3H, CH_3_-5); ^13^C NMR (101 MHz, MeOH-d4) δ 77.47 (C-1), 76.37 (C-3), 75.41 (C-5), 74.07 (C-2), 73.73 (C-4), 18.28 (CH_3_(C-1)), 17.22 (CH_3_(C-5)). HR-MS calcd. for C_7_H_14_NaO_4_^+^: 185.0784; found: 185.0804.

### Competitive binding assay

The competitive binding assay based on fluorescence polarization was performed as described previously [15]. Briefly, 20 µL of a stock solution of LecB (225 nM) and fluorescent reporter ligand N-(fluorescein-5-yl)-N'-(α-l-fucopyranosyl ethylen)-thiocarbamide (1.5 nM) in TBS/Ca (20 mM Tris, 137 mM NaCl, 2.6 mM KCl at pH 7.4 supplemented with 100 µM CaCl2) were mixed with 10 µL serial dilutions (10 mM to 128 nM) of testing compounds in TBS/Ca in triplicates. After addition of the reagents, the microtiter plates were centrifuged at 800 rpm for 1 min at 23°C and incubated for 3 - 5 h at r.t.. Fluorescence emission parallel and perpendicular to the excitation plane was measured on an INFINITE F500 plate reader (Tecan Austria GmbH) or on a PheraStar FS (BMG Labtech, Germany) plate reader with excitation filters at 485 nm and emission filters at 535 nm in black 384-well microtiter plates (Greiner Bio-One, Germany, cat no 781900). On the Tecan instrument, the G-factor was set on 0.92154 and the gain to 80. The measured intensities were reduced by buffer values and fluorescence polarization was calculated. The data were analyzed using BMG Labtech MARS software and/or with Graphpad Prism and fitted according to the four parameter variable slope model. Bottom and top plateaus were defined by the standard compounds l-fucose (**8**) and methyl α-d-mannoside (**2**) respectively and the data was reanalyzed with these values fixed. A minimum of three independent measurements of triplicates each was performed for every ligand.

### Isothermal titration calorimetry (ITC)

LecB was dissolved in TBS/Ca (20 mM Tris, 137 mM NaCl, 2.6 mM KCl at pH 7.3 supplemented with 100 µM CaCl2). The concentration of the monomer of LecB was determined by UV spectroscopy at 280 nm using a molar extinction coefficient of 6990 M^−1^cm^−1^ [24]. The temperature of the sample cell was 25°C. The titration was performed with a solution of ligands **3–7** in the same buffer. ITC was performed on a Microcal ITC200 (General Electric) and the data was analyzed according to the one site binding model using the Microcal Origin software. A minimum of three independent titrations was performed for each ligand. Means and standard deviations are given in the results section. Two independent titrations for 1-deoxy mannose (**5**) were performed as competitive titration in analogy to Turnbull *et al*. [25] with **3** as high affinity ligand and the data were analyzed with least-squares nonlinear regression analysis of the competitive binding model [26] using the Microcal Origin software. In the fitting procedure, thermodynamic parameters of the high affinity ligand **3** were fixed (values used are given in the results section for ligand **3**) and variable parameters were allowed for the low affinity ligand. Arbitrary start values for the low affinity ligand were chosen to initiate the fitting procedure.

### Molecular dynamics (MD) simulations

Molecular dynamics (MD) simulations were done with the AMBER 12 suite of programs [27]. In all simulations the particle mesh Ewald (PME) method [28] was used to treat long-range electrostatic interactions and the SHAKE method [29] to constrain bond lengths of bonds involving hydrogen atoms. The time step was set to 2 fs with a non-bonded cutoff of 9 Å. The protein and calcium parameters were taken from the modified version of the Cornell *et al*. [30] force field (parm99bsc0) and from Li *et al*. [31], respectively. The parameters for the ligands were generated using the antechamber module of AMBER [32]. The ligands were manually sketched using GaussView [33]. Partial charges were calculated with the Gaussian03 [33] program following the Merz-Singh-Kollman scheme [34]. The other parameters of the ligand were obtained from the general amber force field (GAFF) [35]. A pre-equilibrated system including correct solvation was first generated based on the X-ray structure of the tetramer of LecB with l-fucose in all four binding sites (pdb-code 1OXC). From the experimental structure, only the four monomers, the Ca(II) ions and the L-fucose in the first binding site were retained. Crystallographic water molecules, sulfate ions, and the other three fucose molecules were removed. For the first fucose only the coordinates corresponding to the α-anomer were kept. The complex was placed in a periodic truncated octahedron of TIP3P-Ew water molecules [36] and counter ions (Na^+^) were added to maintain electro-neutrality of the system. The borders of the truncated octahedron were chosen to be at least 12 Å from every solute atom. The system was equilibrated by first minimizing 1000 steps to relax unfavourable conformations in the crystal structure or generated by the standard placement of the missing atoms, then heating to 300 K during 200 ps of NVT-MD (constant volume and temperature), and finally relaxing the pressure to 1 bar during 4 ns NPT-MD (constant pressure and temperature). The long pressure adaptation was needed to obtain the correct water density especially at the box boundaries. Harmonic restraints with force constants of 5 kcal mol^−1^ Å^−2^ where applied to all atoms of the complex. These restraints were then gradually reduced to zero during 500 ps of NVT-MD. Production runs were performed for 20 ns (NVT). The same procedure was then repeated for a single α-l-fucose molecule in solution. Corresponding simulations were then performed for all other ligands just by replacing α-l-fucose with the corresponding ligand in the input structure. Even if an X-ray structure of α-d-mannose (pdb-code 1OUR) is available, the simulations for this and the other mannose derivatives were also started from the 1OXC structure. This had to be done since thermodynamic integration (see below) demands for exact matching of the coordinates in the non-changing parts of the systems. Due to the high similarity of the structures 1OXC and 1OUR (see Figure S8 in File S1), only minor influences are expected with respected to the used experimental structure.

The relative binding free energies of α-l-fucose, 1-deoxy l-fucose (**3**), β-l-fucopyranosyl methanol or hybrid (**4**), 1-deoxy d-mannose (**5**), α-d-mannose, methyl α-d-mannoside (**2**), and methyl α-l-fucoside (**1**) were calculated using Thermodynamic Integration [37] by alchemistic transforming the molecules into each other in the binding site of LecB as well as in aqueous solution and subtracting the resulting free energies of these transformations. The pairs of ligands were chosen to have the smallest changing groups possible. The atomic coordinates and box parameters of the pre-equilibrated systems were directly used in these calculations. Two independent runs were performed starting from the snapshot at 1 ns and 2 ns of the simulation of LecB with α-l-fucose, respectively. The protocol of Steinbrecher *et al*. [38] was used with slight modifications. The alchemistic transfer was performed in three steps. First, partial charges on the vanishing groups were removed. Then, the vanishing group was mutated to the appearing group and finally, the charges were added back onto the appearing group. Nine independent simulations of intermediate systems (λ values equally spaced between 0.1–0.9) were run for each of these steps. In the simulations of the 2^nd^ step, in which new atoms were formed, soft potential were used [39]. After 500 steps of energy minimization as well as 200 ps and 400 ps of constrained and unconstrained equilibration, the production runs were 5 ns long. Numerical integration of the δV/δλ values using the trapezoid rule resulted in very similar results for both simulations starting from the different input structure. This can be seen from the small standard deviations calculated by analyzing every ns of the TI simulation production runs independently.

## Results and Discussion

To analyze the individual contributions of the lipophilic C-6 methyl group in **1** and the hydrogen-bonding and lipophilic interacting hydroxymethyl group in **2** to the binding to LecB, the following molecules were designed and synthesized: 1-deoxy l-fucose (**3**), 1-deoxy d-mannose (**5**), β-l-fucopyranosyl methanol (**4**, named hybrid **4** in the following), β-l-fucopyranosyl methane (**6**, named deoxyhybrid **6** in the following) and 1-deoxy d-arabinose (**7**) (Figure 1). The hybrid-type structure **4** was designed to study a potential synergism between the hydrophobic interaction of the fucose-derived equatorial methyl group and the hydrogen bonding of the mannose-derived hydroxymethyl group with Ser23 to the overall binding affinity.

**Figure 2:**
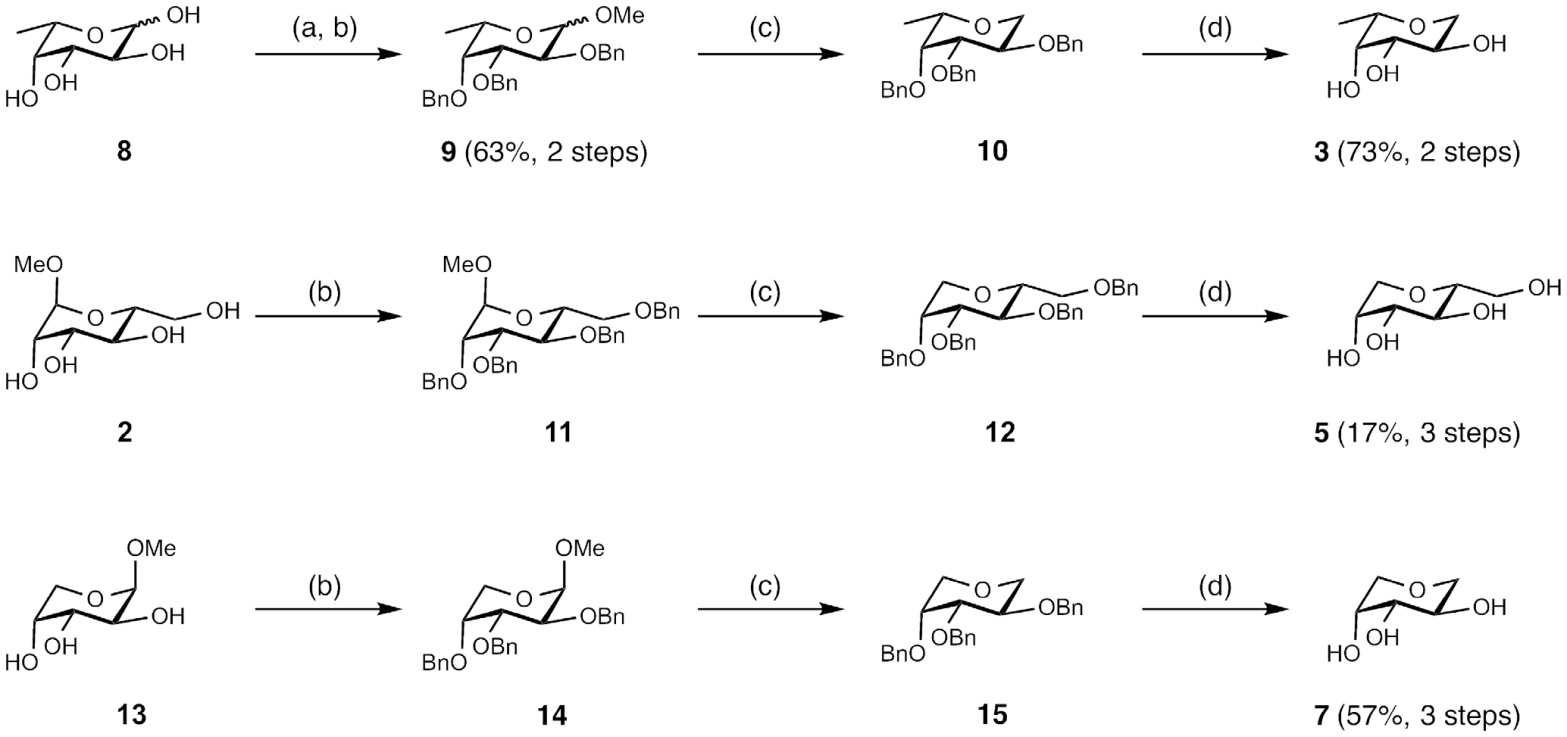
Synthesis of 1-deoxy glycosides 3, 5 and 7. Reagents and conditions: (a) Amberlite IR120 (H^+^), MeOH, 65°C, 1.5 d; (b) NaH, DMF, BnBr, 0°C - r.t., 2 - 12 h; (c) TMSOTf, Et_3_SiH, CH_2_Cl_2,_ 0°C - r.t., 15 h; (d) Pd/C, H_2_, EtOH, r.t., 7 h.

**Figure 3:**
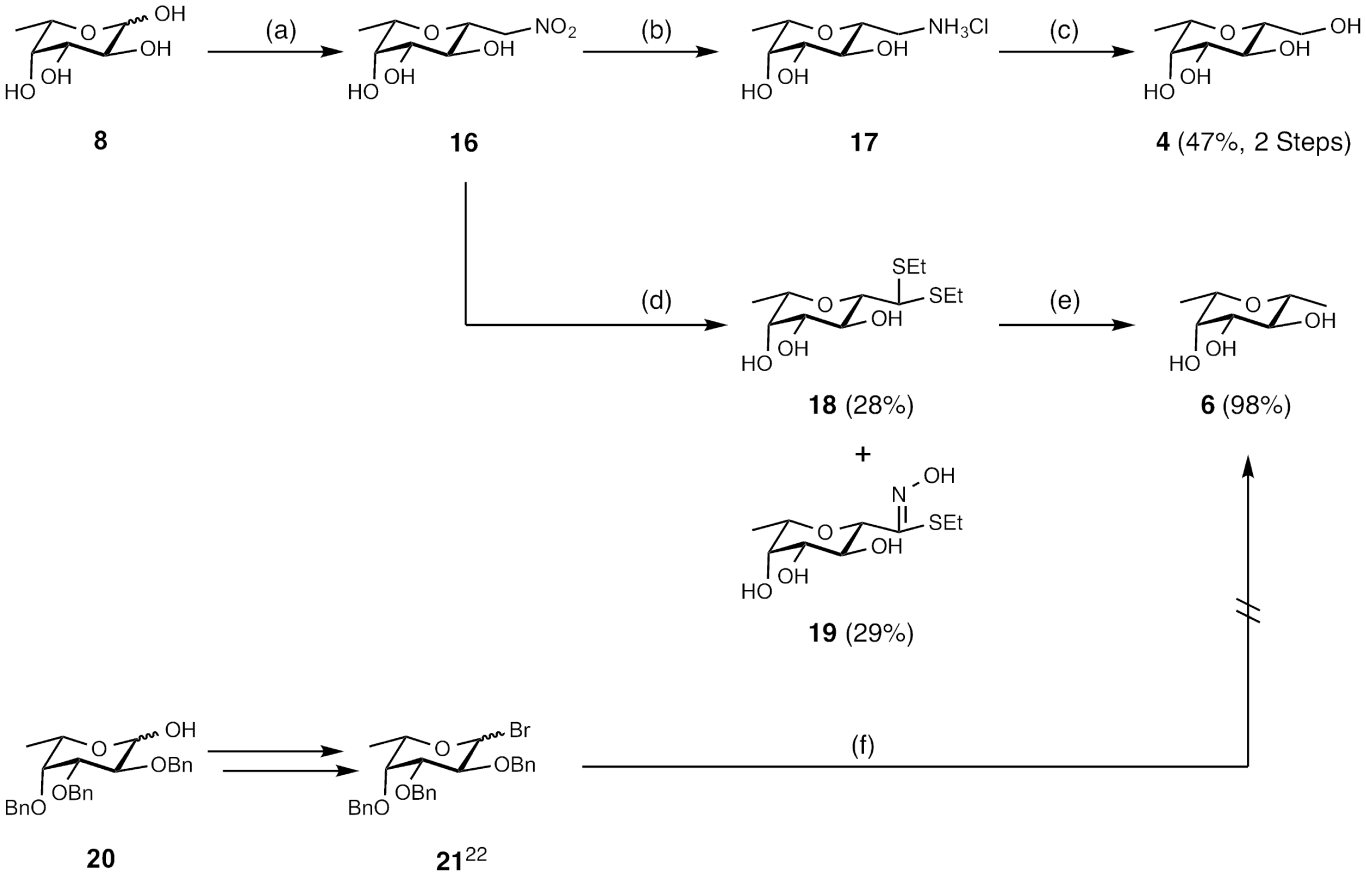
Synthesis of the fucose/mannose hybrid 4 and its derivative deoxyhybrid 6. Reagents and conditions: (a) Compound **16** was synthesized as reported [20]; (b) Pt/C, H_2_, HCl, MeOH, r.t., 2 d; (c) NaNO_2_, AcOH, H_2_O, r.t., 1 d; (d) NaOMe/MeOH, r.t., 30 min, AcCl/EtSH, 0°C, 1.5 h, r.t., 1 h; (e) Raney Nickel, EtOH, 78°C, 30 min; (f) various conditions following the protocol of Bihovsky *et al*. [41].

For the synthesis of deoxygenated fucose **3**, l-fucose (**8**) was glycosylated under Fischer conditions and after benzylation (→**9**) reduced at the anomeric center with triethylsilane and Lewis acid catalysis according to the procedure by Guo *et al*. [19]. In a similar way, 1-deoxy d-mannose (**5**) and 1-deoxy d-arabinose (**7**) were obtained in four chemical steps in high yields from commercially available methyl glycosides **2** and **13**, respectively (Figure 2). The synthesis of the hybrid structure **4** was briefly reported without disclosure of synthetic procedures by Carchon *et al.* [40] starting from d-mannose over five steps. Here, a more efficient approach without the use of protecting groups to yield **4** is described (Figure 3). l-fucose (**8**) was transformed into the Henry adduct **16** using nitromethane and 1,8-diazabicyclo [5.4.0] undec-7-en as a base [20]. Subsequent reduction of the nitro group in **16** to amine **17** with hydrogen over Pt/C as catalyst, further diazotation under acidic aqueous conditions with NaNO_2_ followed by hydroxylation in one pot yielded **4** in 47% over two steps.

To access methyl C-glycosides or cis-2,6-dimethyltetrahydropyrans (as present in **6**) by S_N_2 reaction of halides, a high β-selectivity using organocuprates was reported by Bihovsky *et al*. [41]. However, we were unable to synthesize C-fucoside **6** by nucleophilic substitution of glycosyl bromide **21** [22] (Figure 3). Neither an increase of the organocuprate reagent Me_2_CuLi nor elevated reaction temperatures (−78°C – r.t.) yielded the desired C-glycoside. Increasing the reactivity of nucleophiles with organo-lithium reagents MeLi or MeLi/TMEDA [42] was also unsuccessful. Petruš and co-workers [43] reported an alternative strategy for the synthesis of methyl C-gluco- and galactosides by denitration *via* the Nef reaction with *in situ* dithioacetal formation and subsequent desulfurization with Raney Nickel. Therefore, we transformed β-l-fucopyranosyl nitromethane (**16**) according to the reported procedure for gluco- and galactosides, but only S-ethyl-N-hydroxythioimidate **19** and untransformed **16** were recovered. By inverting the order of addition of the reagents, diethyl dithioacetal **18** could be obtained in 28% yield, and unreacted **16** as well as smaller amounts of the side product **19** (29%) were recovered. After desulfurization with Raney Nickel the desired compound **6** was isolated in 98% yield.

**Figure 4:**
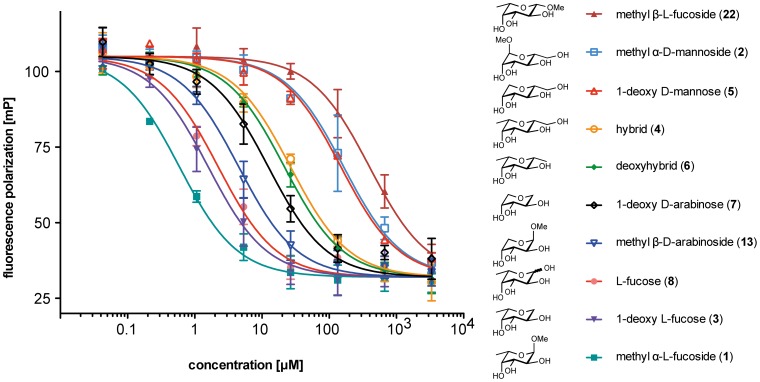
Evaluation of the complete set of LecB ligands described in this study for competitive binding to LecB using a fluorescence polarization-based assay. By fitting a one site binding model, IC_50_ values were obtained (values are depicted in Figure 5). Here, one representative titration of triplicates is shown and error bars were determined by triplicates on one plate. Average IC_50_ values (see Figure 5) and standard deviations were determined from three independent measurements of triplicates each.

**Figure 5:**
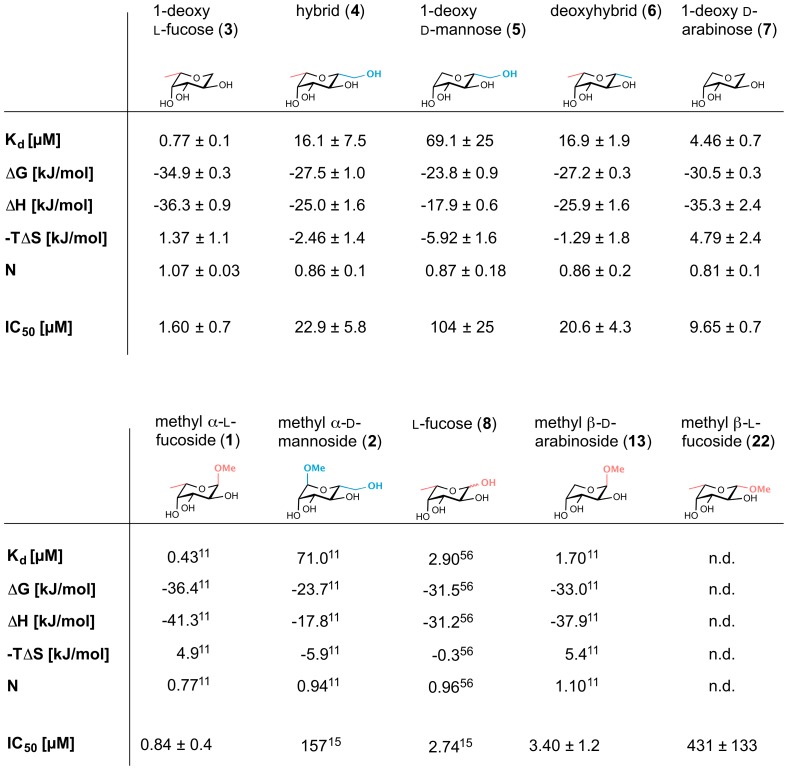
Biophysical evaluation of LecB-binding to ligands of this study: comparison of isothermal microcalorimetry (ITC) with competitive binding assay. Means and standard deviations were determined from minimum three independent titrations or from minimum three independent measurements of the competitive binding assay. Binding thermodynamics for 5 were determined by indirect titration with 3 as high affinity ligand in two independent titrations. ITC data for 1, 2, 8 and 13 and IC_50_ values for 2 and 8 were taken from the references indicated [11], [15], [56]. n.d.  =  not determined.

Subsequently, the interactions of compounds **3–7** with LecB were analyzed in a previously developed competitive binding assay [15] based on fluorescence polarization (Figure 4, Figure 5). Furthermore, a set of related and literature-known LecB ligands was evaluated: methyl α-l-fucoside (**1**), methyl α-d-mannoside (**2**), l-fucose (**8**), methyl β-d-arabinoside (**13**). In addition, methyl β-l-fucoside (**22**, Figure 4) was analyzed as a ligand with an isomeric equatorial substituent, which is unable to establish the hydrogen bond with Ser23 as observed for **2** in the crystal structure. The IC_50_ values of the latter compounds were in good agreement with the previously published dissociation constants (see Figure 5). From the competitive binding assay, the affinities of all tested compounds could be grouped: fucose derivatives **1**, **3**, and **8** showed the highest affinity with IC_50_ values ranging from 840 nM to 2.7 µM, arabinose derived ligands **13** and **7** showed IC_50_s of 3.4 and 9.7 µM, mannose derivatives **2** and **5** had IC_50_s of 157 and 104 µM and the LecB ligands combining properties from both fucose and mannose, hybrid **4** and deoxyhybrid **6** showed IC_50_ values in an intermediate affinity range with IC_50_s of 23 and 21 µM, respectively. Furthermore, methyl β-l-fucoside (**22**) was assayed and a strongly reduced binding affinity of 431 µM was measured.

**Figure 6:**
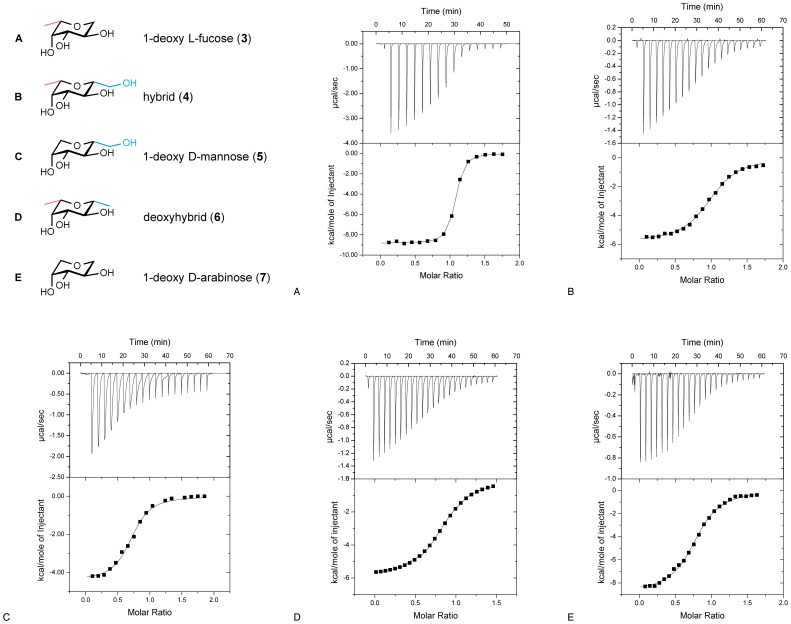
Isothermal microcalorimetry of LecB with novel ligands 3–7 without glycosidic properties. By fitting a one site binding model, thermodynamic data of the interactions were obtained (values are depicted in Figure 5). Here, one representative titration of independent triplicates is shown. In contrast to the direct titration of LecB with high affinity ligands **3**, **4**, **6**, and **7**, binding thermodynamics for the weak binder **5** were determined by indirect titration with **3** as high affinity ligand in two independent titrations.

The interactions of 1-deoxy l-fucose (**3**), hybrid **4**, 1-deoxy d-mannose (**5**), deoxyhybrid **6**, and 1-deoxy d-arabinose (**7**) with LecB were further studied by isothermal microcalorimetry to characterize their thermodynamic dissociation constants and thermodynamic fingerprints (Figure 5, Figure 6). The dissociation constants obtained were in good agreement with data from the competitive binding assay. The glycosidic linkages in **1** and **2** had only a small impact to binding as observed from their 1-deoxy analogs **3** and **5**, respectively: upon removal of the glycosidic oxygen and the methyl aglycon, K_d_ values increased by a factor of 1.8 for methyl α-l-fucoside (**1**). This corresponds to a difference in Gibbs energy of −1.5 kJ/mol, which could result from the loss of a H_2_O-mediated hydrogen bond in 1-deoxy l-fucose (**3**), previously reported [44] for the high resolution crystal structure of **1** with LecB. In case of 1-deoxy mannose (**5**) the binding affinity is nearly identical, because the glycosidic methyl group has no direct contact to the protein as deduced from the crystal structure of the complex. For 1-deoxy d-arabinose (**7**), the loss of the glycosidic linkage resulted in a slightly stronger decrease in binding affinity compared to its parent glycoside **13**, presumably due to increased entropic costs of the less stabilized chair conformation in **7** as a result of the reduced number of equatorial substituents in comparison to **3** and **5**.

Methyl α-l-fucoside (**1**) and methyl β-d-arabinoside (**13**), a derivative lacking the lipophilic methyl group, differ in their thermodynamic binding affinity by a factor of four [11]. Comparable data (difference in K_d_ 4- to 6-fold) was obtained by microcalorimetry of hybrid **4** and 1-deoxy d-mannose (**5**), as well as for 1-deoxy l-fucose (**3**) and 1-deoxy d-arabinose (**7**) indicating the magnitude of the contribution of the equatorial methyl group in fucosides to the binding affinity for LecB. Deduced from comparable contributions of the equatorial methyl groups, these three independent sets of results suggest similar binding modes of the polyhydroxylated tetrahydropyrans **3** to **7** to those determined in the crystal structure of the monosaccharides **1** and arabinoside **13** [11] with LecB.

We thus confirmed that the unusual high affinity of LecB for **1** partially results from the equatorial methyl group and quantified its contribution. Since mannoside **2** forms an additional hydrogen bond *via* O-6 with Ser23 of the protein in the crystal structure of the complex, we combined both moieties in one molecule, *i.e.*, hybrid **4** (Figure 1). Contrary to an anticipated synergism of binding effects in **4**, its interaction with LecB (K_d_ 16.1 µM, IC_50_ 22.9 µM), was only fourfold stronger than mannoside **2** (K_d_ 71 µM, IC_50_ 157 µM), but significantly weaker than fucoside **1** (K_d_ 0.43 µM, IC_50_ 0.84 µM). To asses steric repulsion for this unexpectedly low affinity, the isomeric structure of **4**, methyl β-l-fucoside (**22**), unable to establish a hydrogen bond with Ser23 of the receptor, was compared to its α-anomer **1** for binding to LecB. In good agreement to the ELLA-based analysis by Wu *et al.* [45], we observed a 500-fold reduction in affinity in the competitive binding assay for the β-anomer **22** compared to its α-anomer **1**, suggesting a steric and/or electrostatic repulsion from the equatorial methoxy substituent in **22**. Surprisingly, we observed a comparable binding affinity between deoxyhybrid **6** and the presumably hydrogen-bond forming hybrid **4**. These comparable dissociation constants, in combination with the comparable corresponding enthalpic and entropic contributions to the binding, suggest a low population and low contribution to the binding affinity of this hydrogen bond with Ser23 in solution, which had been observed in the crystalline state at low temperatures for mannose.

**Figure 7:**
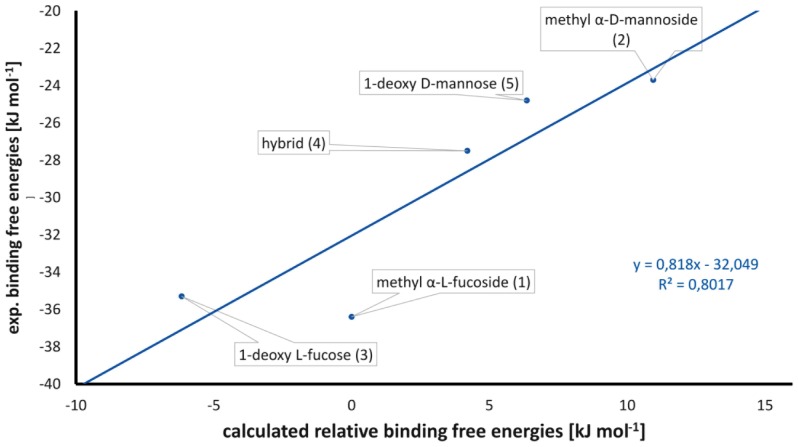
Correlation between calculated and experimental binding free energies.

**Figure 8:**
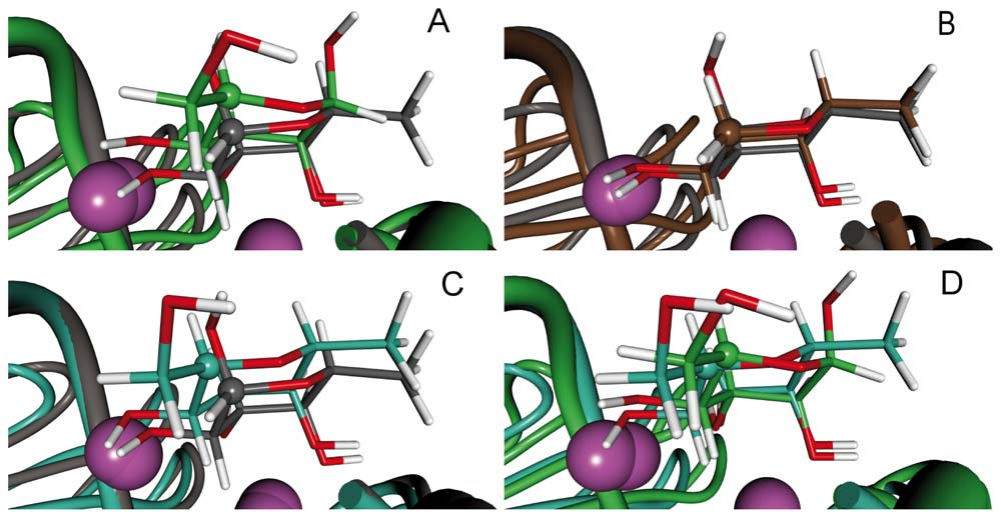
Overlays of representative structures (ns 6-15 of the molecular dynamics simulations) are shown for four LecB-ligand complexes. The atom corresponding to C1 of fucose (or C5 of mannose, respectively) is marked as ball in the capped-sticks representation. (A) α-l-fucose (grey) and α-d-mannose (green), (B) α-l-fucose (grey) and 1-deoxy l-fucose (3, brown), (C) α-L-fucose (grey) and hybrid (4, cyan), (D) α-d-mannose (green) and hybrid (4, cyan). The calcium ions are shown as purple spheres. The complexes were aligned on the binding site residues (21–24, 45, 95**–**104 of the first and 114 of the second monomer).

In general a significant reduction in binding affinity was observed, when equatorial substituents were introduced at the superimposing fucose-C1- or mannose-C5-position or their derivatives, *e.g.*, compounds **2**, **4**, **5**, and **6**. In order to understand the steric and electrostatic reasons for this decrease of binding affinity when such an equatorial substituent is present, molecular dynamics (MD) simulations were performed. Thermodynamic integration, [37], [38] a highly accurate method to predict binding free energies, was used to calculate the relative affinities of 7 pairs of ligands (Table 1). Such calculations are based on the idea of alchemistic modification of one structure into another one. For fast convergence, these alchemistic structural changes should be as small as possible and the pairs were chosen accordingly. Relative binding free energies were approximated by summing over all single transitions leading from methyl α-l-fucoside (**1**) to the specific ligand, that means **1** was chosen as arbitrary zero point of the energy scale. Generally, a very good agreement of theoretical values with experimental binding affinities (Figure 7) was obtained. A good correlation coefficient (R^2^ = 0.8) was obtained with a good separation between the high- and low-affinity ligands. When looking at the independent steps (Table S1 in File S1) of the TI simulation of the transition from 1-deoxy l-fucose (**3**) to its equatorially substituted derivative **4**, *i.e.*, the simulation separating the two groups of ligands, it becomes evident that the loss in binding affinity is caused by an unfavorable steric fit and that this effect is reduced by stronger electrostatic interactions. Because the force-field parameters used gave good descriptions of the energetic features of binding, we performed extended simulations (20 ns) on specific complexes to analyze the structural basis for the differences in binding affinities (Figure 8). All complexes adopt very similar orientations of the ligands, which was further visualized by analyzing the time series of root mean square deviations (RMSD) of all ligand atoms compared to the X-ray structure of l-fucose. RMSDs are stable over the simulation time around 1 to 2 Å (Figure S1 in File S1). However, a significant difference can be seen in the pose of the low-affinity ligands compared to the high-affinity derivatives. Upon equatorial substitution of the carbon atom corresponding to C1 of fucose, the sugar moiety in the complexes of LecB with α-d-mannose (Figure S2 in File S1) or hybrid **4** (Figure S3 in File S1) is pushed out from the binding site by approx. 0.7 Å, due to unfavorable steric interactions of the equatorial hydroxymethyl substituent with Asp96.

**Table 1:** Relative binding free energies calculated using thermodynamic integration.

Entry	Ligand Pair	Change in Binding Free Energy [kJ/mol]	Standard Deviation [kJ/mol]
1	α-l-fucose	−2.12	0.17
	1-deoxy l-fucose (**3**)		
2	1-deoxy l-fucose (**3**)	10.37	1.63
	hybrid (**4**)		
3	hybrid (**4**)	2.15	2.24
	1-deoxy d-mannose (**5**)		
4	1-deoxy d-mannose (**5**)	7.16	2.14
	α-d-mannose		
5	α-d-mannose	−2.57	1.91
	methyl α-d-mannoside (**2**)		
6	α-L-fucose	4.05	0.40
	methyl α-l-fucoside (**1**)		

The values are averages over two independent calculations of 5 ns length. For the calculation of the standard deviation, each ns simulation time was analyzed independently.

**Figure 9:**
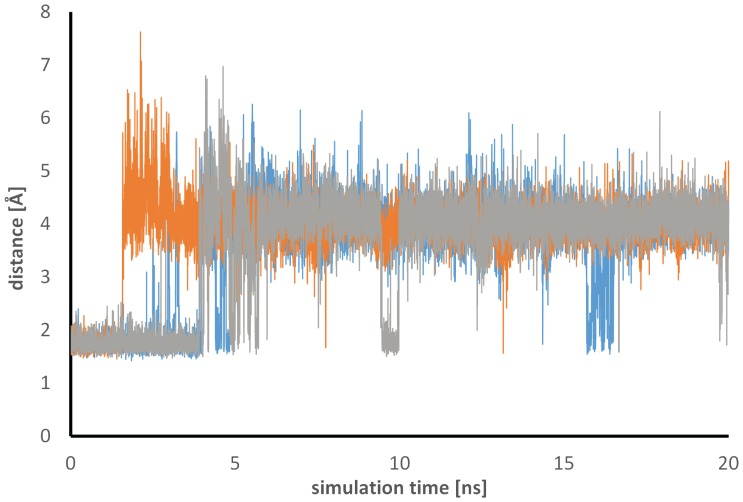
The hydrogen bond between Ser22 and Asp96 is disturbed by α-d-mannose. Time series of the shortest distance between HG of Ser22 and OD1/OD2 of Asp96 of three independent simulations (grey, orange, blue) of the complex of LecB with α-d-mannose.

In the crystal structure of the complex of l-fucose (**8**) with LecB, the ligand is interacting with Asp96 by its atom HO2. Asp96 is highly restrained to its position due to formation of a large hydrogen-bond network with other residues (Figure S4 in File S1). Addition of a β-substituent results in a steric clash with the protein, which is reduced in the simulations of the complex of both **4** and α-d-mannose by the outward shift of the ligand (Figure 8A, C). In the simulation of α-d-mannose, this repulsive interaction is additionally avoided by a conformational change of the protein and Asp96 flips away from the β-substituent after 4 ns simulation time (Figures S5b and S6 in File S1). This change is accompanied by the breakage of the hydrogen-bond network in the protein as seen in the time series of the distance between Asp96 and Ser22 (Figure 9). This new position is, however, not optimal and additional transitions between the conformation with and without the hydrogen bond, called closed and open form in the following, occur during the simulations. Representative structures of the closed (ns 1–4) and open (ns 11–15 of the simulation) form are shown in Figure S8 in File S1. Both show ligand poses reasonably close to the X-ray structures with the open form having a slightly larger RMSD (see time series of the ligand all-atom RMSD in Figure S1 in File S1). RMSD values around 1.5 Å are seen for the open form compared to values around 1.0 Å for the closed form. l-Fucose shows even smaller RMSD values of 0.7 Å (see also Figure S1 and Figure S8 in File S1). This difference is probably related to the fact that the l-fucose X-ray structures was taken as starting point and should, therefore, not be over-interpreted since the differences in the two X-ray structures are in this range. Two additional independent simulations of the complex of α-d-mannose with LecB confirm the result that the open conformation is adopted with the higher probability interrupted by short periods when the hydrogen bond is formed (see Figure 9 and Figure S2 in File S1).

**Figure 10:**
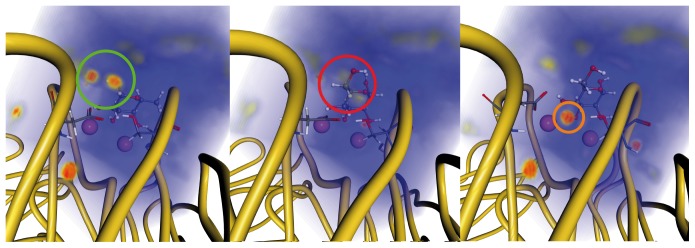
Water density around the ligands in the complex of LecB with l-fucose (left), and d-mannose (closed form: middle, open form: right). Low, medium and high probability to find a water oxygen atom at a specific position is color coded in blue, yellow and red, respectively. The calcium ions are shown as purple spheres.

Besides direct interactions between ligands and protein, changes in the solvent structure will also influence binding affinities. It is, however, not possible to split the affinities obtained with thermodynamic integration into different contributions as this can be done, *e.g.*, using MM/PBSA or MM/GBSA [46]–[48] and, thus, the influence of the solvents cannot be directly extracted from these calculations. Instead, we analyzed the solvation structure and thermodynamics around the ligand using the Grid Inhomogeneous Solvation Theory (GIST). [49]–[51] Such calculations create a three-dimensional mapping of the properties on a rectangular grid by analyzing the positional and orientational preferences of the solvent molecules taken from explicit solvent simulation data. Snapshots from the first simulation of α-d-mannose representing the closed (ns 2–5) and open (ns 12–15) form were analyzed. Additionally, the α-l-fucose simulation (ns 12–15) was used for comparison. Here, we concentrated on the water density maps depicting favorable interactions of solute and solvent molecules. The α-l-fucose complex shows two regions of high water density close to Asp96, Ser22, and O2 of fucose (green cycle, Figure 10). The water molecules complete the hydrogen-bonding network discussed above and favor complex formation. In the closed form of the mannose complex, this solvent structure is not observed and few regions with a slightly increased solvent density (red cycle, Figure 10) appeared. In the open form a highly structured water molecule is visible bridging the distance between the two amino acids Asp96 and Ser22 (orange cycle, Figure 10).

Interestingly, the first simulation run with hybrid **4** only showed the closed form. To validate these contradicting results, two additional simulations were performed now also showing transitions between the open and the closed form (Figure S7 in File S1). Thus, interchange between the open and closed form is possible in the complex of **4** with LecB, however, with a perhaps different probability as for the complex of α-d-mannose with LecB. This phenomenon could originate from the additional methyl group in **4**, which stabilizes the complex by van der Waals interaction with the protein. The importance of the van der Waals interaction of fucose with Thr45 in LecB has previously been analyzed by Mishra *et al.* [52] In the same study, a similar flexibility of loops in the carbohydrate recognition domain, as observed here for the low affinity ligand, was reported in absence of the high affinity ligand fucose. Although the conformation of LecB is generally conserved in the low-temperature crystal structures deposited in the protein data bank, flexibility at ambient conditions seems thus reasonable supporting our observations. To further validate these results, we are currently working on an experimental proof for the flexibility and rearrangement of the protein conformation.

## Conclusion

We have dissected the contribution of individual substituents of the natural ligands fucosides (*e.g.*, **1**) and mannosides (*e.g.*, **2**) to binding with LecB. The lipophilic interaction of the methyl group of fucose and derivatives increases binding affinity by a factor of 4–6 compared to analogs lacking this methyl group. A combination substituents presumably forming attractive interactions from **1** and **2** with LecB into hybrid **4**, *i.e.*, the equatorial methyl group of fucose and the hydroxymethyl group of mannose, improved binding affinity with respect to mannosides, but surprisingly did not lead to a synergistic effect in binding affinity. Although the hydroxymethyl group in **2** forms an additional hydrogen bond with the lectin in the crystalline low temperature state, our thermodynamic data for **4** and its deoxygenated analog **6** suggest only a minor contribution to binding in aqueous solution. In addition, we conclude from our experimental data and theoretical calculations, that the steric demand of equatorial substituents at the superimposing positions of fucose-C1 and mannose-C5 leads to unfavorable steric interactions with Asp96 and to a high destabilization of the protein surroundings, and in this way, accounts for the reduced affinity of mannosides and derivatives with respect to fucosides.

In the LecB orthologs RS-IIL [53] and BclA [54], a mutation in the so-called sugar specificity loop [55] (Asn21-Ser22-Ser23) of Ser22 in LecB to alanine results in an electrostatic void within the protein, which is filled by mannose O-6 and establishing of the hydrogen bond with Asp96. This explains the higher selectivity of these orthologs for mannose over fucose in contrast to LecB. In case of LecB, the additional hydrogen bond of α-d-mannose with Ser23 observed in the crystal structure, and the entropic gain due to the higher flexibility of Asp96 are not sufficient to compensate for the destabilizing effect induced by equatorial substituents, and consequently result in a reduced binding affinity. Since the β-substituent opens up opportunities to exploit additional binding sites as shown in our previous work [15], new substituents able to take over the structural role of Asp96 are required for future drug design.

## Supporting Information

File S1 **Supporting files.** Data S1, ^1^H- and ^13^C-NMR spectra of new compounds 3, 4, 6, 7, 18, 19. Data S2, Individual titration curves from competitive binding assay. Data S3, Individual titration curves from isothermal calorimetry experiments. Figure S1, Time series of the root mean square deviation (RMSD) of all ligand atoms in the simulation of three complexes of LecB. Before the RMSD calculations the snapshots were aligned based on the binding site residues. Figure S2, Time series of the root mean square deviation (RMSD) of all ligand atoms in the three independent simulation of three complexes of LecB with α-d-mannose. Before the RMSD calculations the snapshots were aligned based on the binding site residues. Figure S3, Time series of the root mean square deviation (RMSD) of all ligand atoms in the three independent simulation of three complexes of LecB with hybrid 4. Before the RMSD calculations the snapshots were aligned based on the binding site residues. Figure S4, Full hydrogen-bond network of Asp96 with Ser22, Gln26, Asp104, and α-l-fucose. The calcium ions are shown as purple spheres. Figure S5, Orientation of Asp96 in the representative structure of the simulations of four complexes of LecB: (A) α-l-fucose (closed), (B) α-d-mannose (open), (C) 1-deoxy l-fucose (3, closed), (D) hybrid 4 (closed); The hydrogen bonds between Asp96 and Ser22 as well as Asp96 and the ligand are indicated by red dashed lines. The calcium ions are shown as purple spheres. Figure S6, Time series of the shortest distance between HG of Ser22 and OD1/OD2 of Asp96 of simulations of three complex of LecB. Figure S7, Time series of the shortest distance between HG of Ser22 and OD1/OD2 of Asp96 of three independent simulations of the complex of LecB with hybrid 4. Figure S8, Overlays of experimental structures and representative structures from the MD simulations: The first structure is shown as a green ribbon, with green carbon atoms and with Ca(II) ions in magenta. The second structure is color coded in grey with brown Ca(II) ions. Upper left: X-ray structure of the fucose (1oxc) and mannose (1our) complex; Upper right: X-ray (1oxc) and MD structure of the fucose complex; Lower left: X-ray structure (1our) and closed form of the mannose complex; Lower right: X-ray structure (1our) and open form of the mannose complex. Table S1, Relative binding free energy for the transition from 1-deoxy l-fucose (3) to hybrid 4 calculated using thermodynamic integration and its partitioning into the free individual steps. The values for two independent calculations of 5 ns length as well as the averages are given.
(PDF)
