# Supplementary material for: A Biophysical Study with Carbohydrate Derivatives Explains the Molecular Basis of Monosaccharide Selectivity of the Pseudomonas aeruginosa Lectin LecB
Source: PLoS One. 2014 Nov 21;9(11):e112822. doi: 10.1371/journal.pone.0112822 (PMC4240550; doi:10.1371/journal.pone.0112822)
Supplement: File S1 — Supporting files. Data S1, 1H- and 13C-NMR spectra of new compounds 3, 4, 6, 7, 18, 19. Data S2, Individual titration curves from competitive binding assay. Data S3, Individual titration curves from isothermal calorimetry experiments. Figure S1, Time series of the root mean square deviation (RMSD) of all ligand atoms in the simulation of three complexes of LecB. Before the RMSD calculations the snapshots were aligned based on the binding site residues. Figure S2, Time series of the root mean square deviation (RMSD) of all ligand atoms in the three independent simulation of three complexes of LecB with α-d-mannose. Before the RMSD calculations the snapshots were aligned based on the binding site residues. Figure S3, Time series of the root mean square deviation (RMSD) of all ligand atoms in the three independent simulation of three complexes of LecB with hybrid 4. Before the RMSD calculations the snapshots were aligned based on the binding site residues. Figure S4, Full hydrogen-bond network of Asp96 with Ser22, Gln26, Asp104, and α-l-fucose. The calcium ions are shown as purple spheres. Figure S5, Orientation of Asp96 in the representative structure of the simulations of four complexes of LecB: (A) α-l-fucose (closed), (B) α-d-mannose (open), (C) 1-deoxy l-fucose (3, closed), (D) hybrid 4 (closed); The hydrogen bonds between Asp96 and Ser22 as well as Asp96 and the ligand are indicated by red dashed lines. The calcium ions are shown as purple spheres. Figure S6, Time series of the shortest distance between HG of Ser22 and OD1/OD2 of Asp96 of simulations of three complex of LecB. Figure S7, Time series of the shortest distance between HG of Ser22 and OD1/OD2 of Asp96 of three independent simulations of the complex of LecB with hybrid 4. Figure S8, Overlays of experimental structures and representative structures from the MD simulations: The first structure is shown as a green ribbon, with green carbon atoms and with Ca(II) ions in magenta. The second structure is col [file pone.0112822.s001.pdf]

Supporting information to

A biophysical study with carbohydrate derivatives explains the molecular basis of monosaccharide selectivity of the *Pseudomonas aeruginosa* lectin LecB

Roman Sommer[1,2], Thomas E. Exner[2,3], Alexander Titz[1,2]*

[1]Helmholtz Institute for Pharmaceutical Research Saarland (HIPS), Campus C 2.3, D-66123 Saarbrücken, Germany

[2]Department of Chemistry and Graduate School Chemical Biology, University of Konstanz, D-78457 Konstanz, Germany

[3]Theoretical Medicinal Chemistry and Biophysics, Institute of Pharmacy, University of Tübingen, D-72076 Tübingen, Germany

* corresponding author

email: alexander.titz@helmholtz-hzi.de

Data S1: ^{1}H- and ^{13}C-NMR spectra of new compounds 3, 4, 6, 7, 18, 19

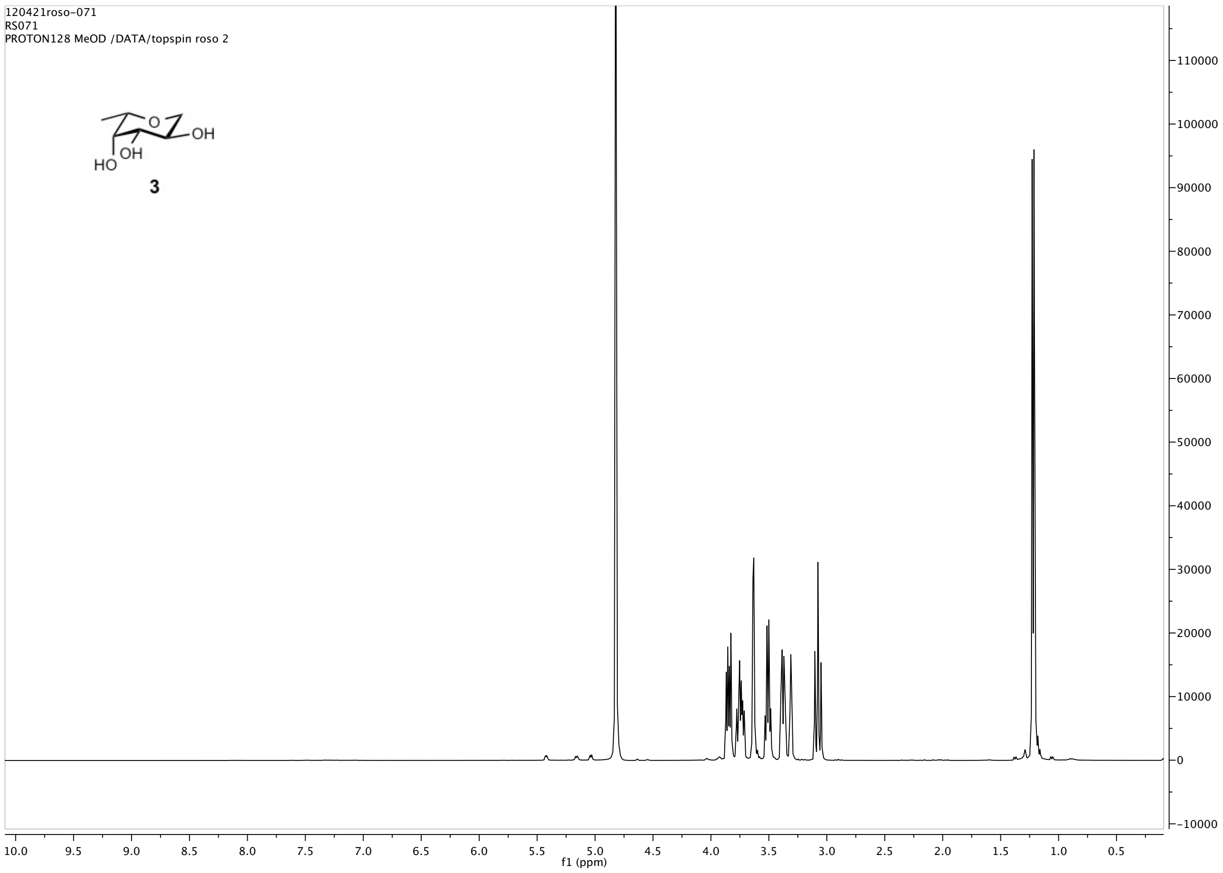

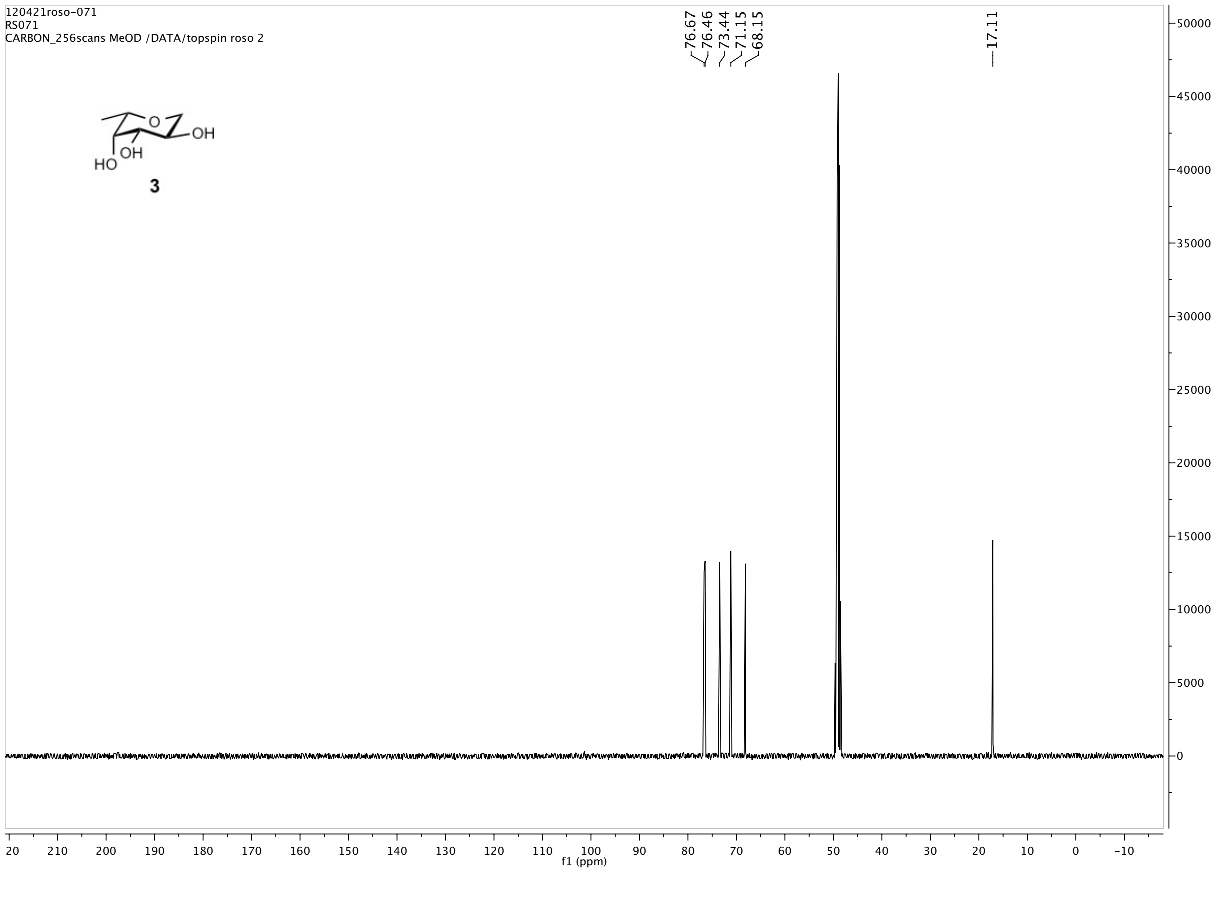

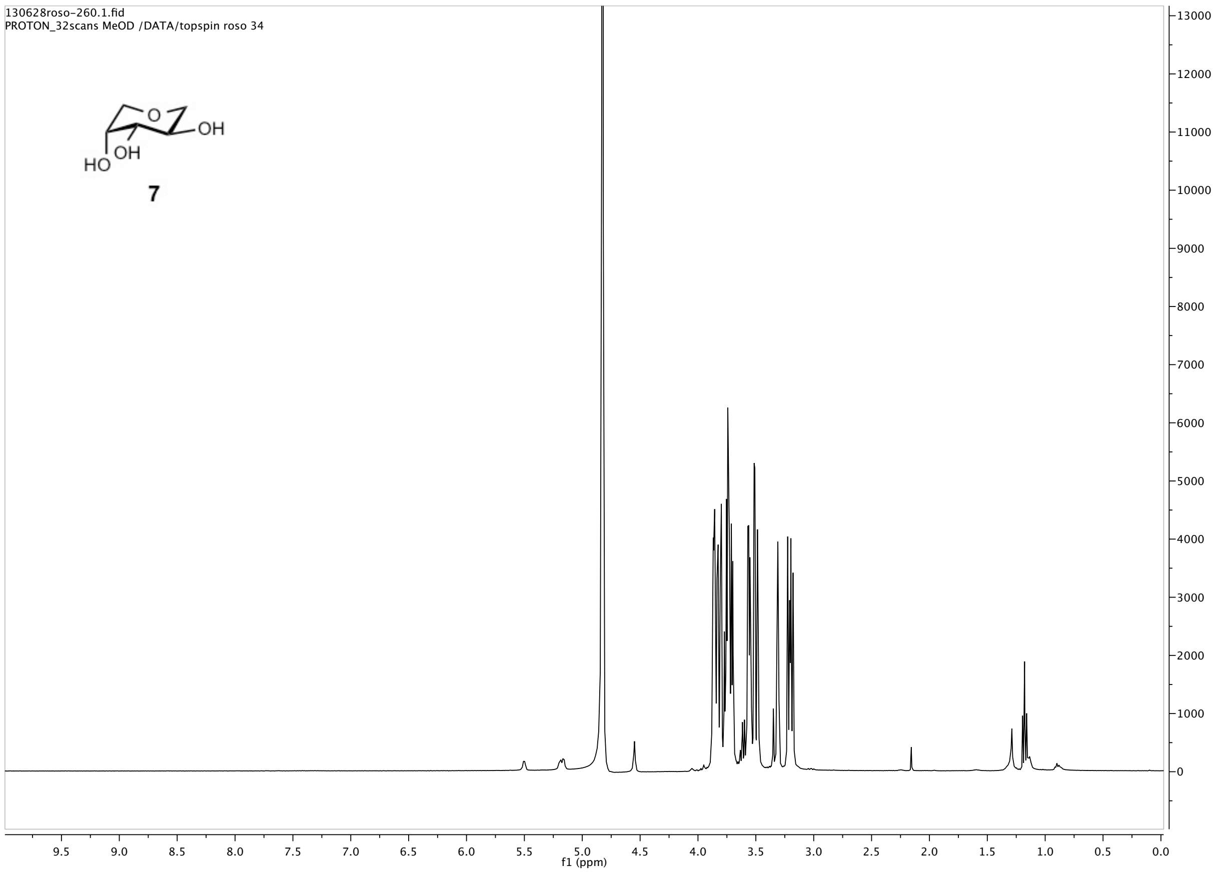
130628roso-260.1.fid
PROTON_32scans MeOD /DATA/topspin roso 34
OH
HO
OH
O
7
f1 (ppm)

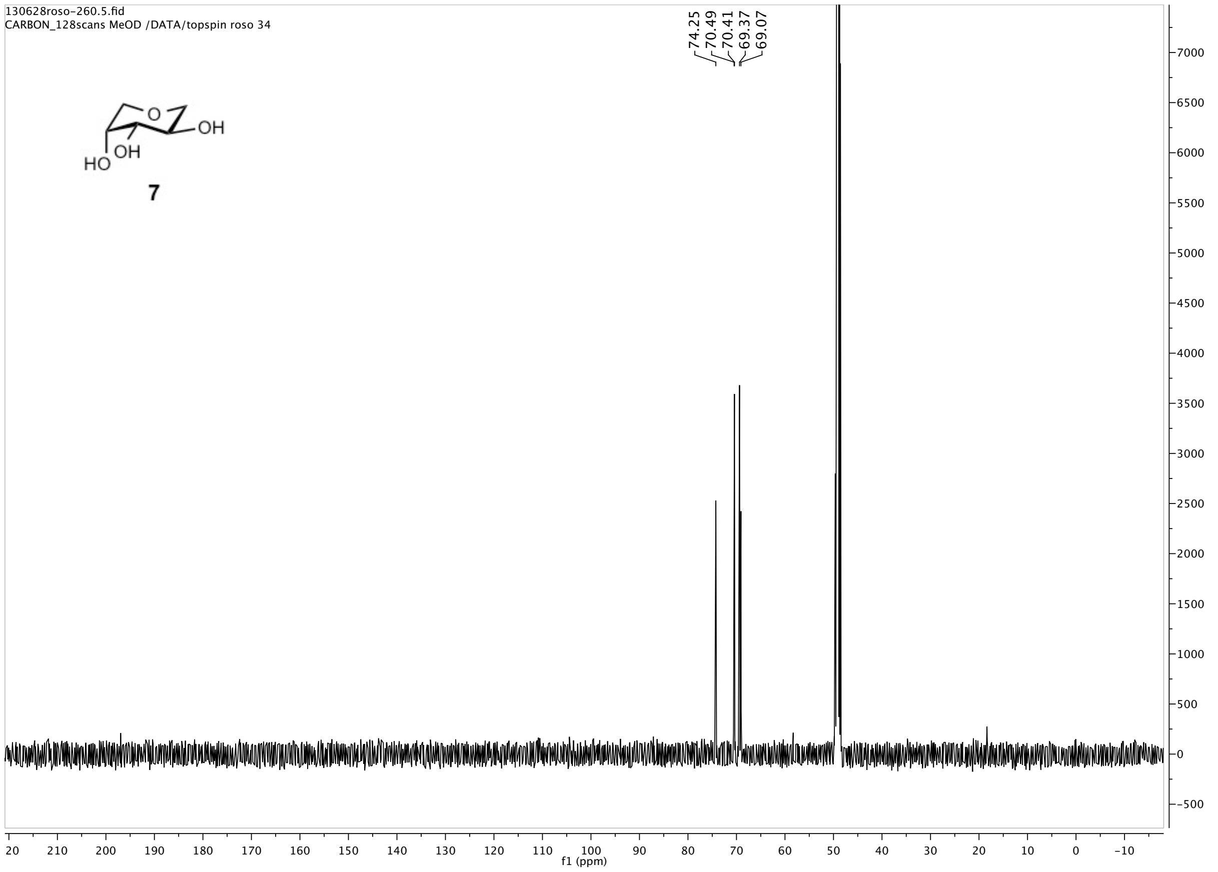
130628roso-260.5.fid
CARBON_128scans MeOD /DATA/topspin roso 34
74.25
70.49
70.41
69.37
69.07
OH
HO
OH
O
7
f1 (ppm)

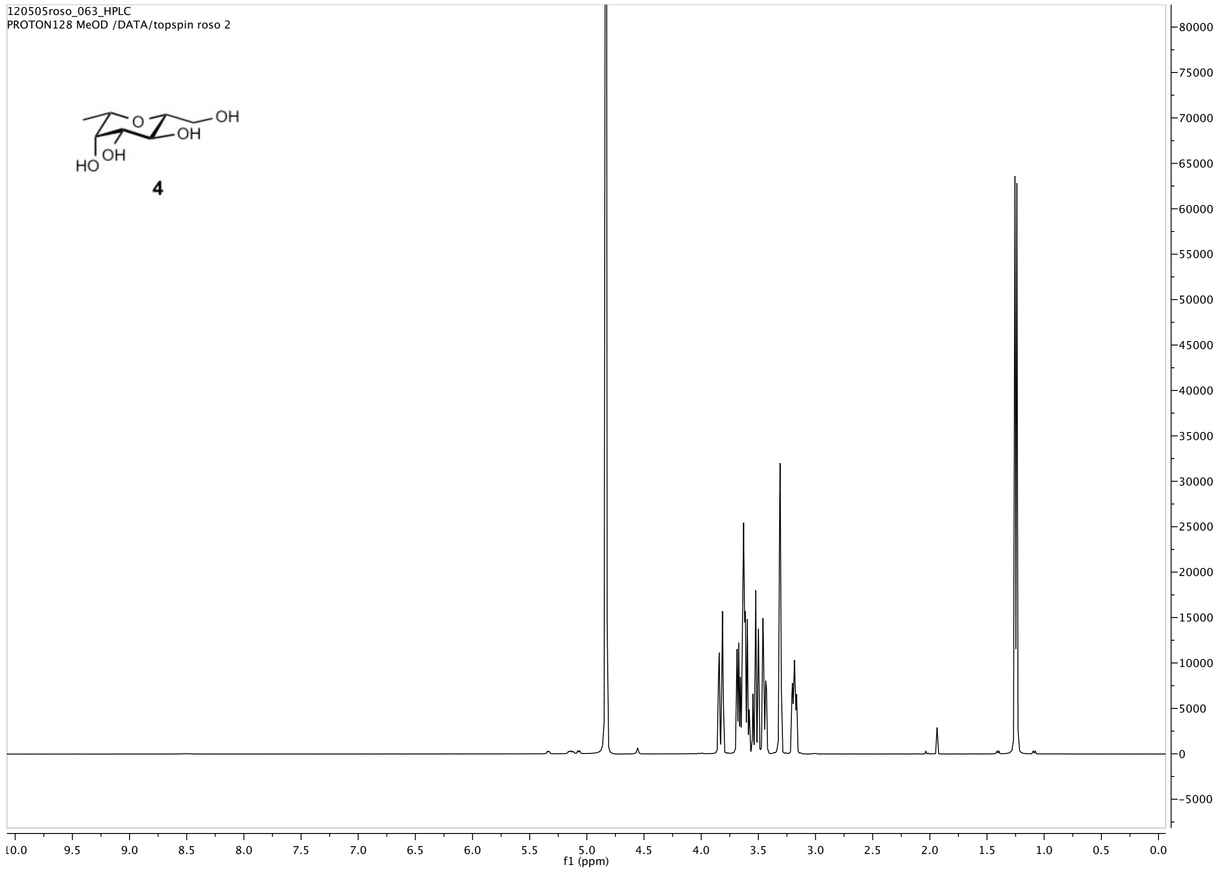
120505roso_063_HPLC
PROTON128 MeOD /DATA/topspin roso 2
4
f1 (ppm)

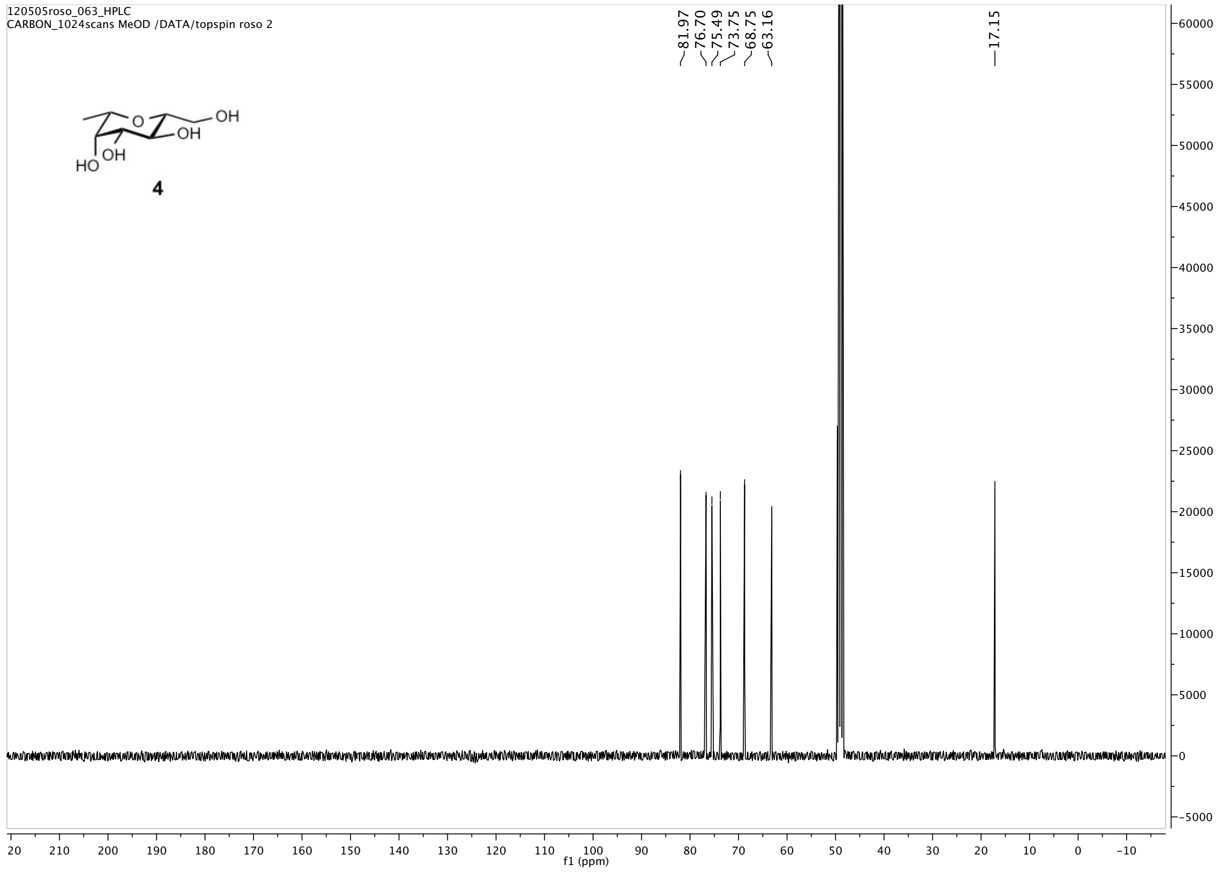
120505roso_063_HPLC
CARBON_1024scans MeOD /DATA/topspin roso 2
81.97
76.70
75.49
73.75
68.75
63.16
17.15
4
f1 (ppm)

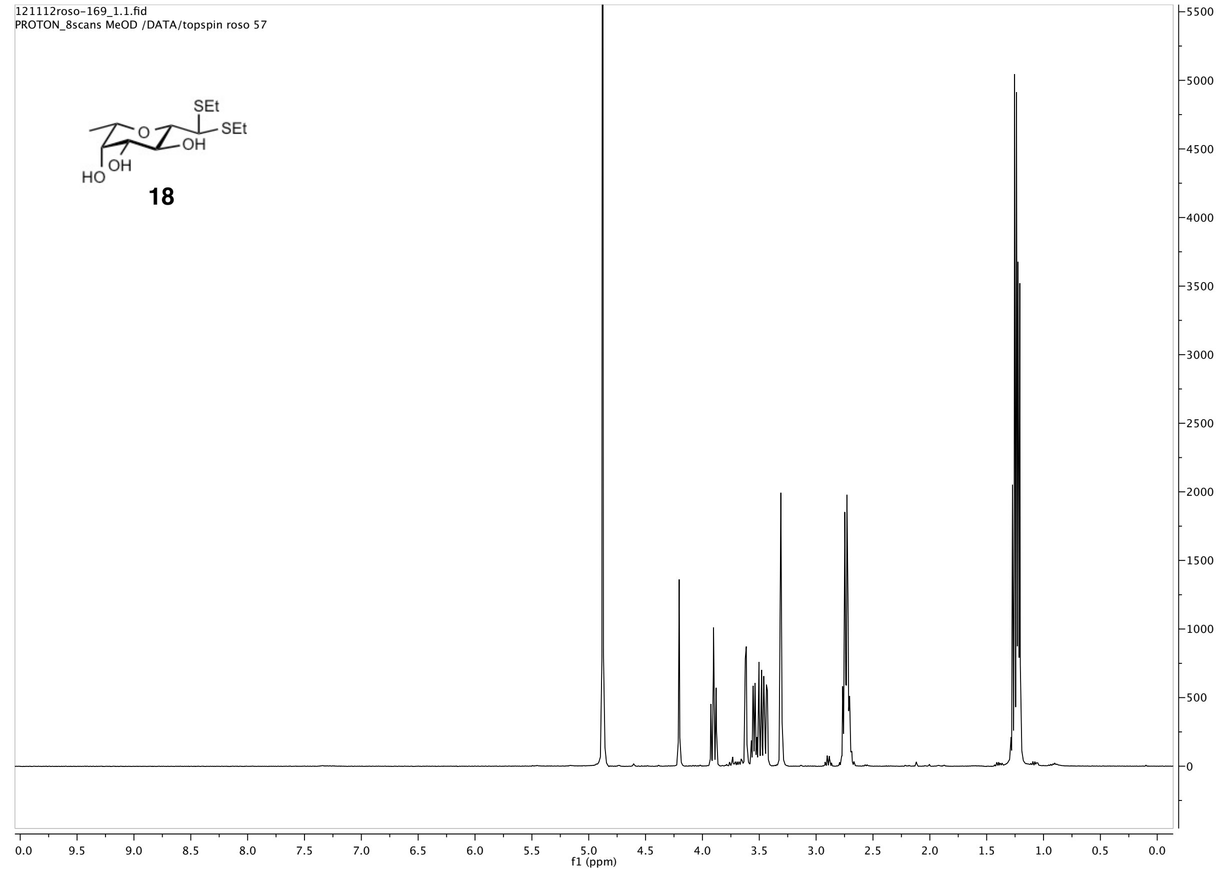
121112roso-169_1.1.fid
PROTON_8scans MeOD /DATA/topspin roso 57
SEt
SEt
O
OH
OH
HO
18
f1 (ppm)

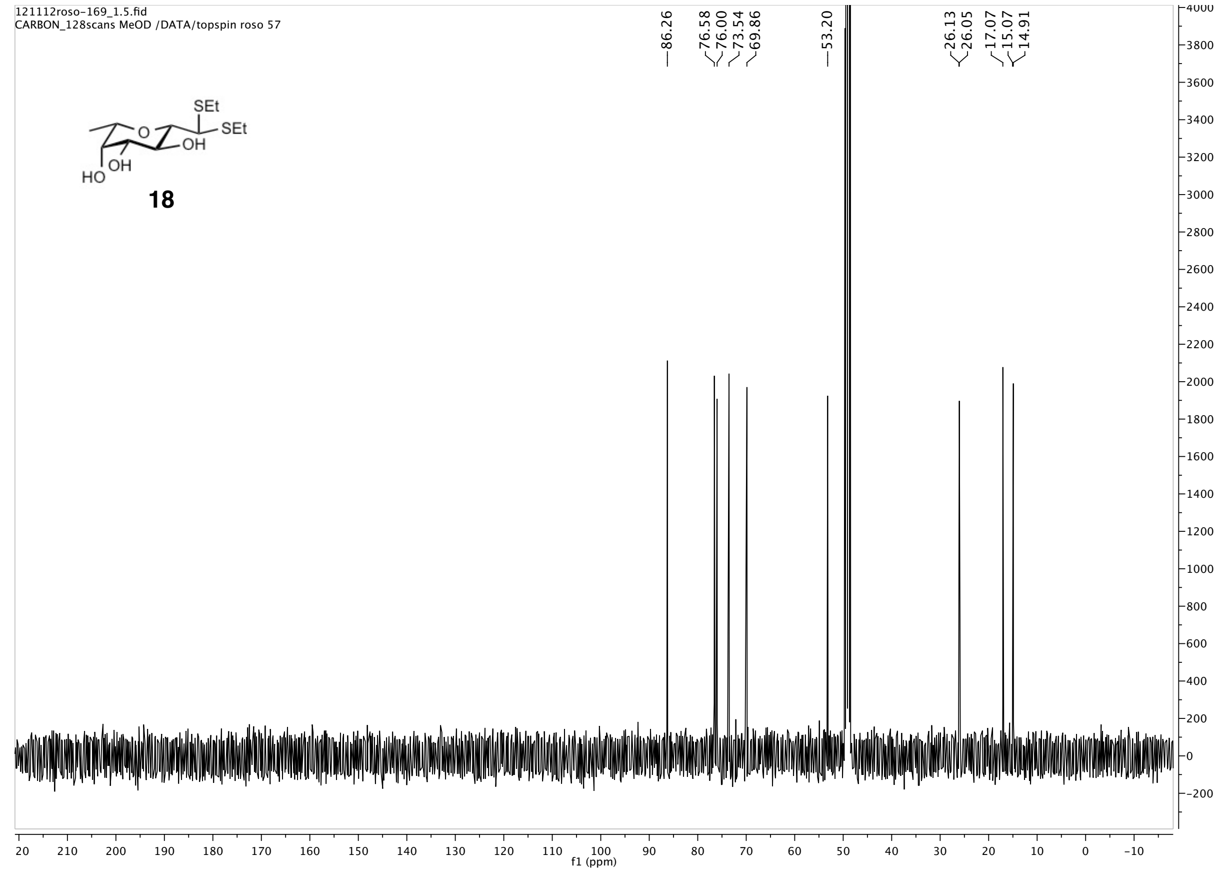
121112roso-169_1.5.fid
CARBON_128scans MeOD /DATA/topspin roso 57
86.26
76.58
76.00
73.54
69.86
53.20
26.13
26.05
17.07
15.07
14.91
SEt
SEt
O
OH
OH
HO
18
f1 (ppm)

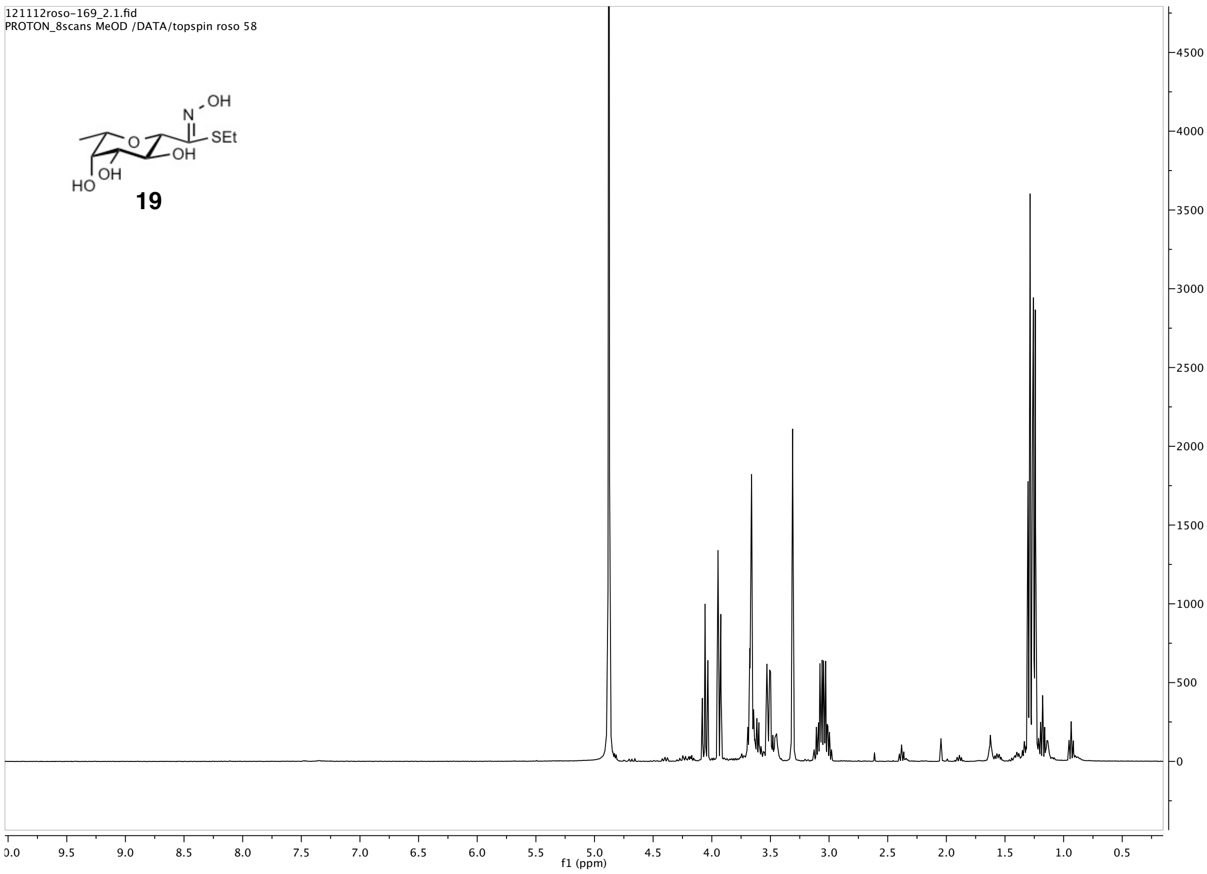
121112roso-169_2.1.fid
PROTON_8scans MeOD /DATA/topspin roso 58
19
f1 (ppm)

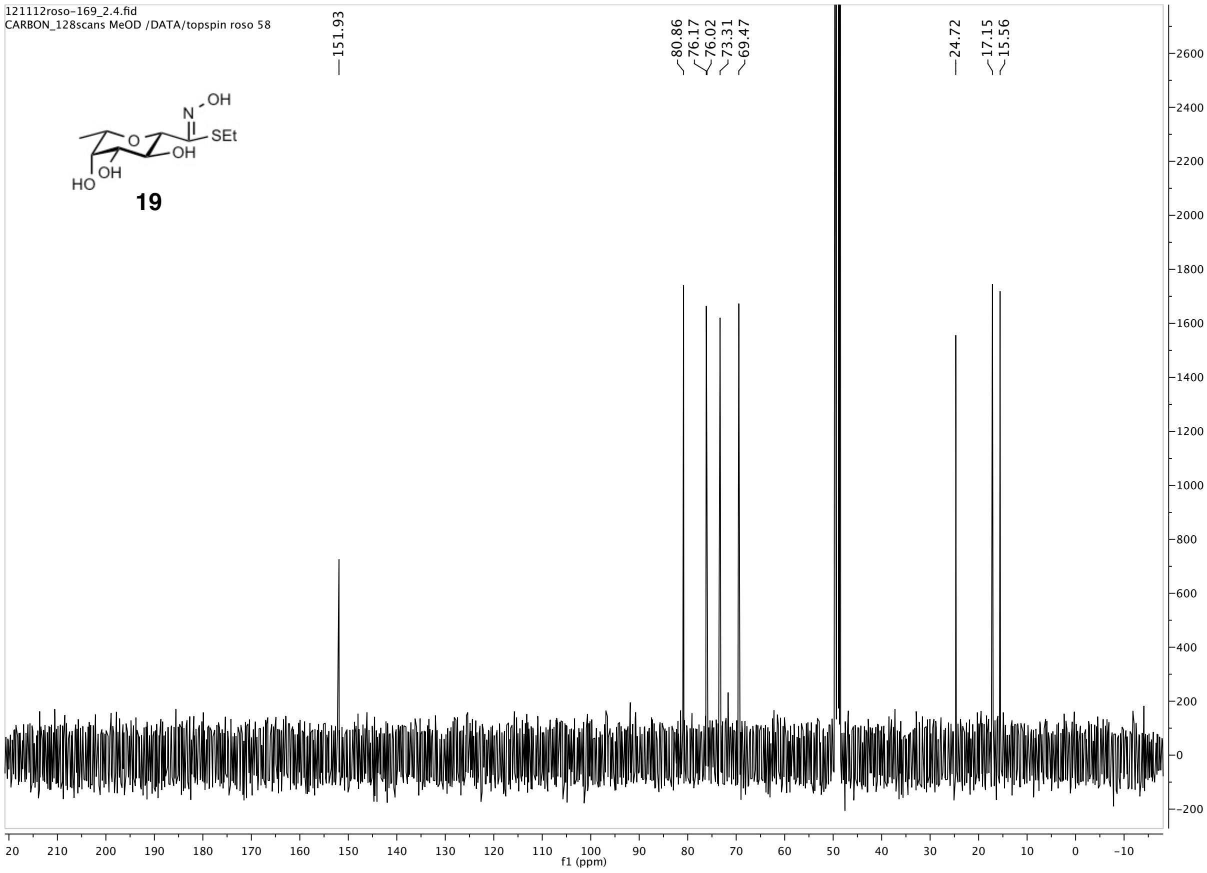
121112roso-169_2.4.fid
CARBON_128scans MeOD /DATA/topspin roso 58
151.93
80.86
76.17
76.02
73.31
69.47
24.72
17.15
15.56
19
f1 (ppm)

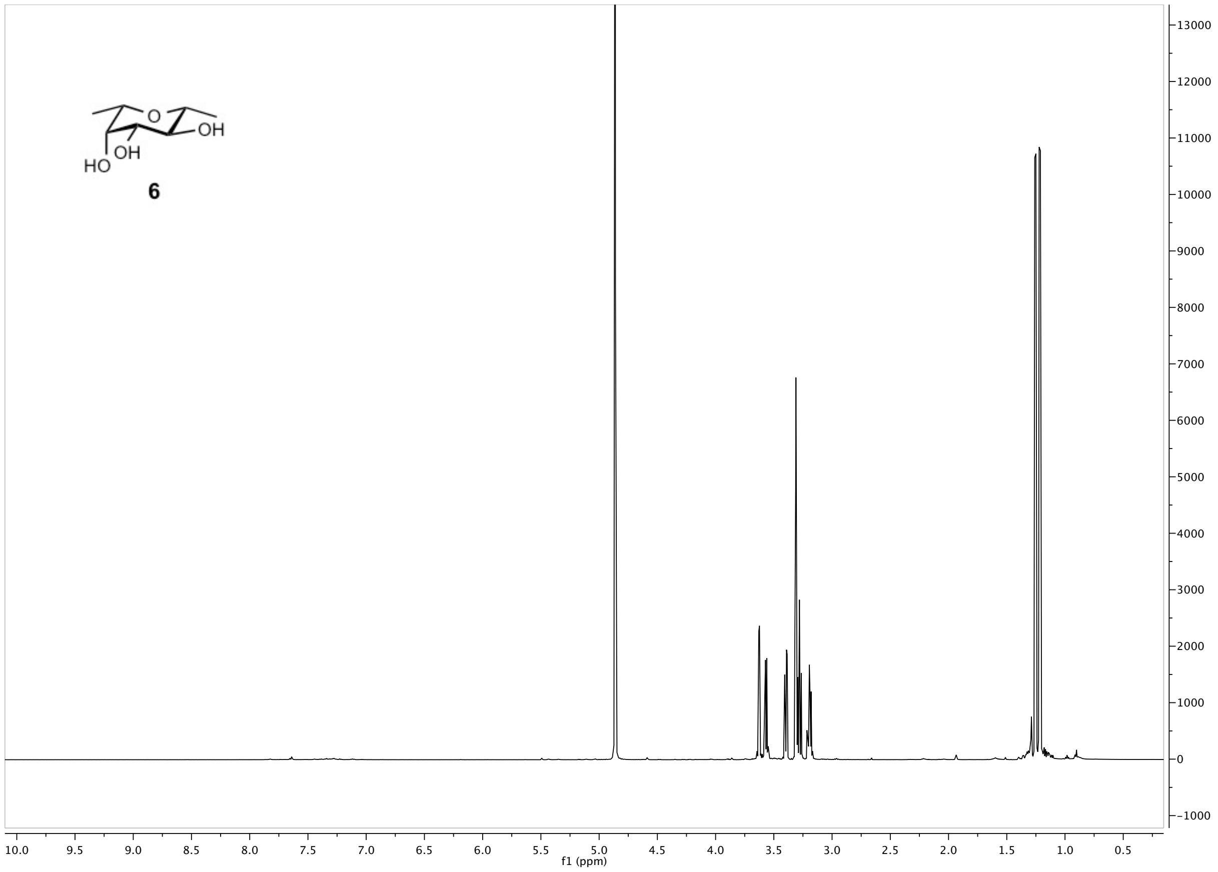
OH
HO
6
f1 (ppm)

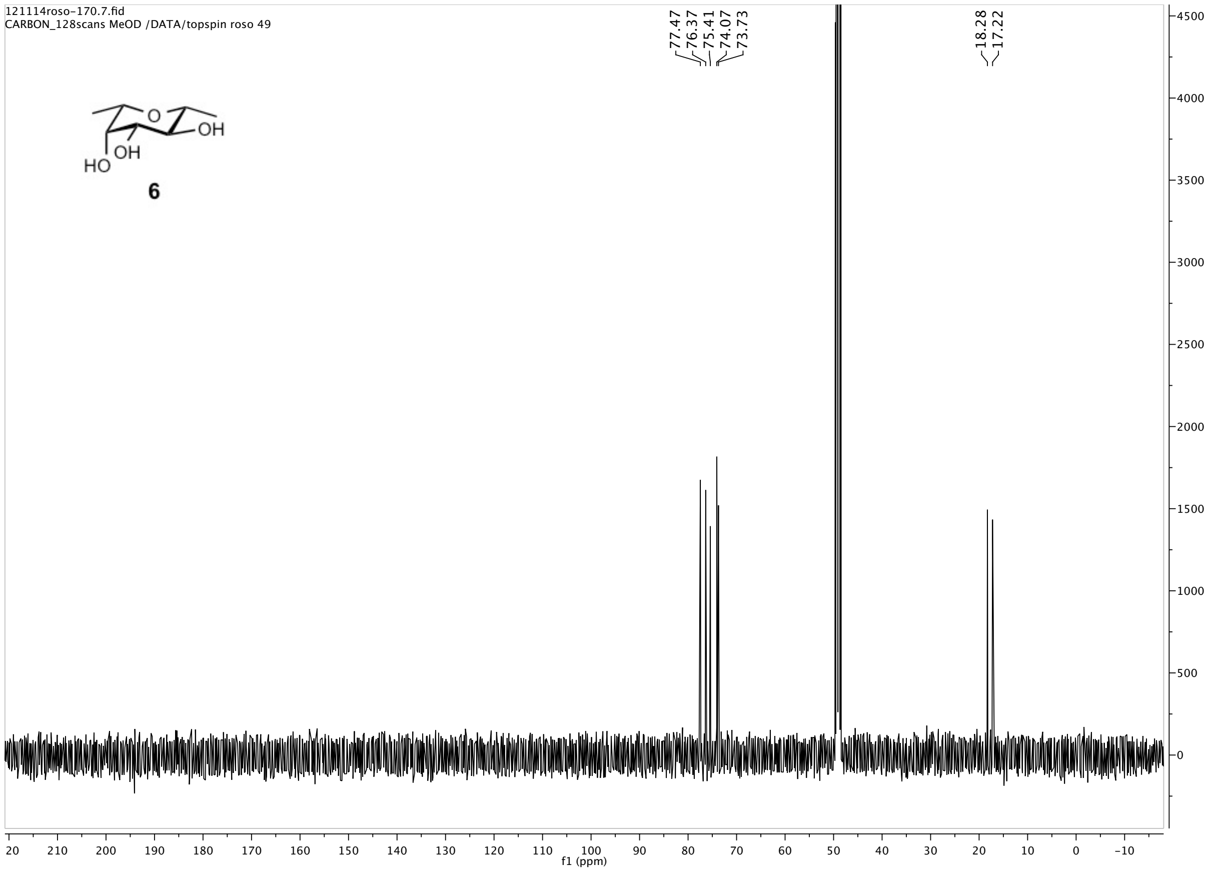
121114roso-170.7.fid
CARBON_128scans MeOD /DATA/topspin roso 49
77.47
76.37
75.41
74.07
73.73
18.28
17.22
OH
HO
6
f1 (ppm)

Data S2: Individual titration curves from competitive binding assay

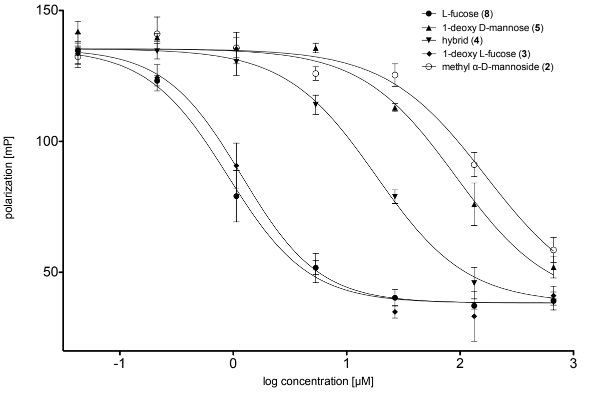

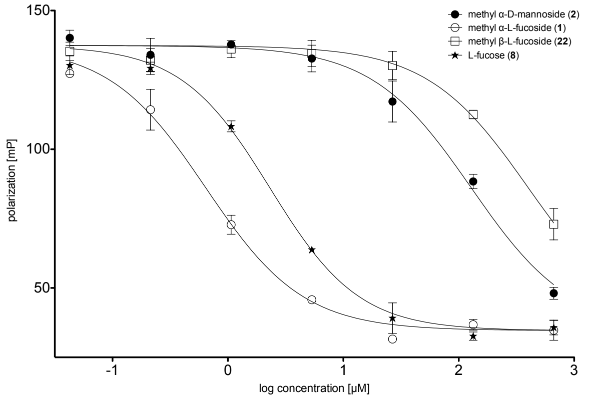

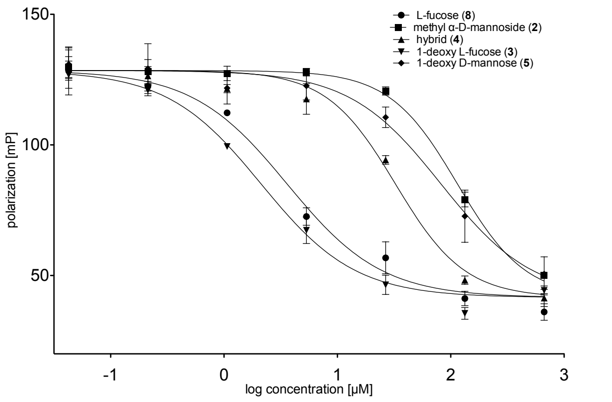

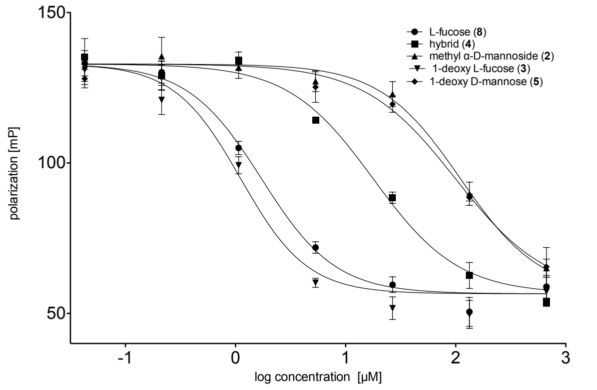

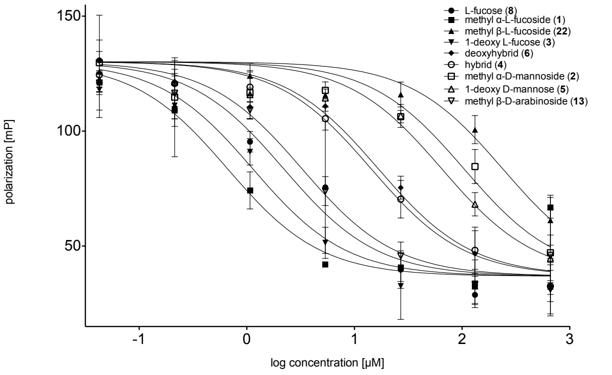

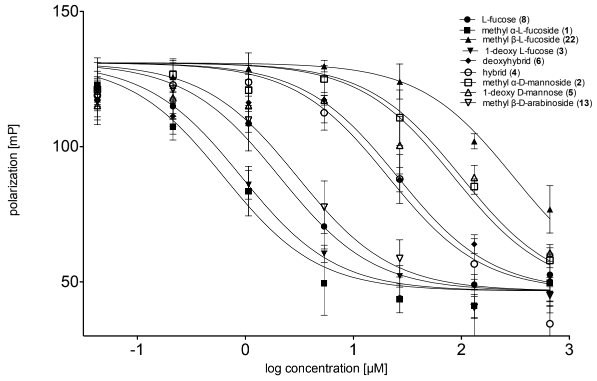

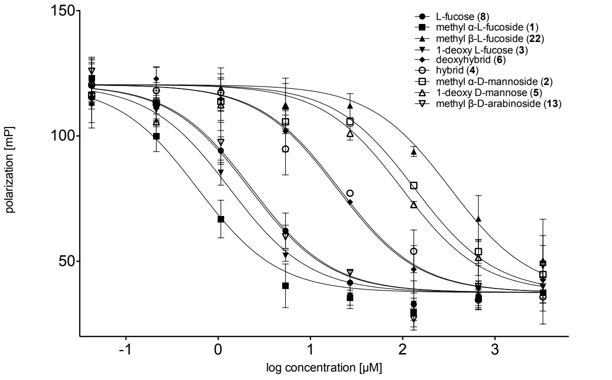

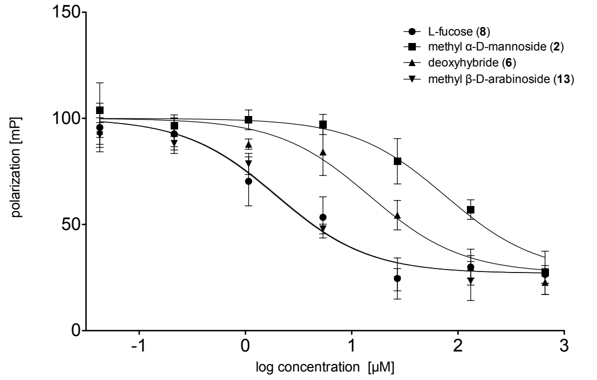

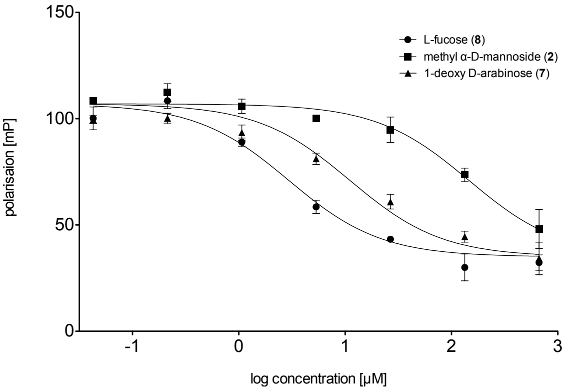

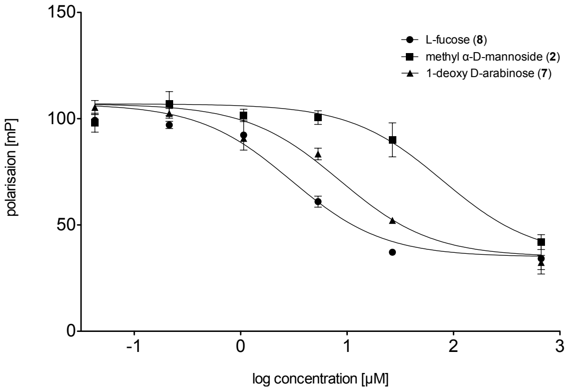

Data S3: Individual titration curves from isothermal calorimetry experiments

1-deoxy L-fucose (**3**) (4 mM) with LecB (224 μM) 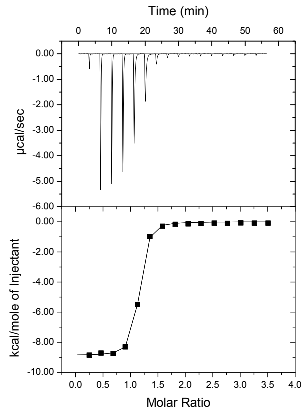	1-deoxy L-fucose (**3**) (2 mM) with LecB (224 μM) 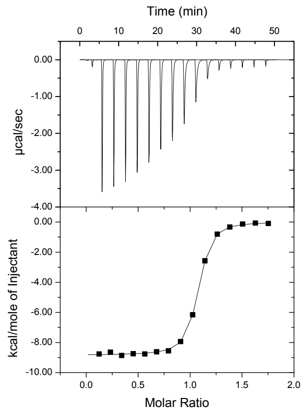	1-deoxy L-fucose (**3**) (390 μM) with LecB (34.3 μM) 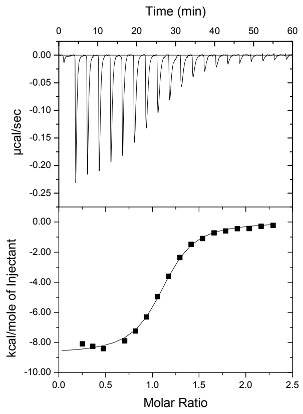
1-deoxy L-fucose (**3**) (380 μM) with LecB (34.3 μM) 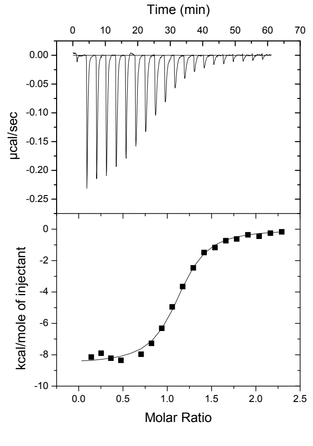	hybrid (**4**) (2.84 mM) with LecB (237 μM) 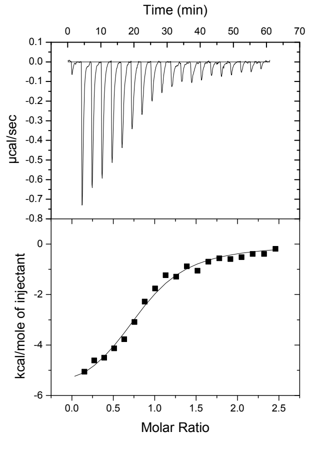	hybrid (**4**) (2.00 mM) with LecB (237 μM) 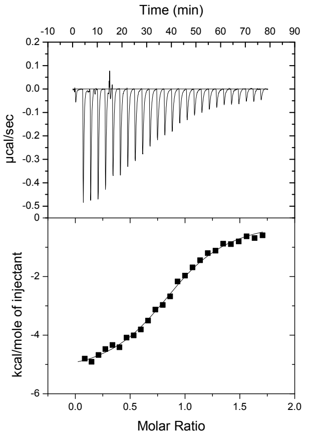

hybrid (**4**) (2.84 mM)
with LecB (343 μM)

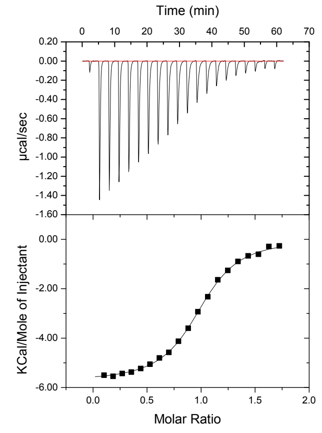

hybrid (**4**) (2.84 mM)
with LecB (343 μM)

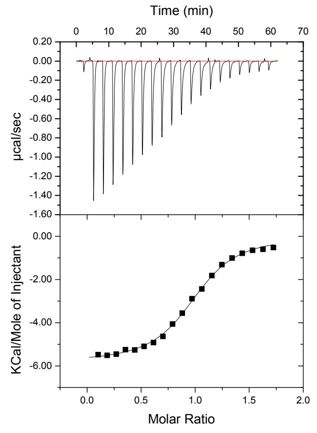

hybrid (**4**) (6.0 mM)
with LecB (681 μM)

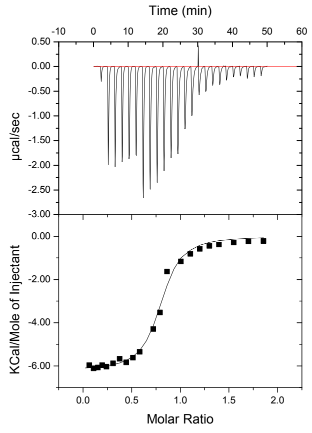

hybrid (**4**) (6.0 mM)
with LecB (681 μM)

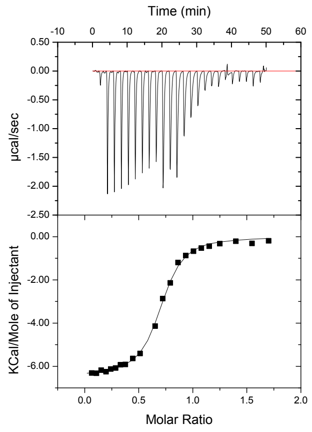

hybrid (**4**) (6.25 mM)
with LecB (681 μM)

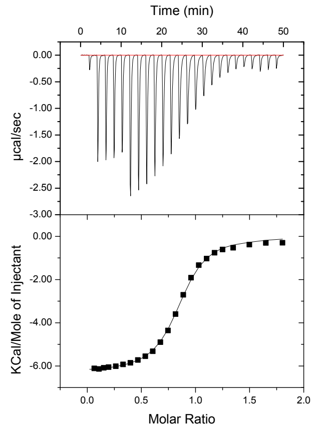

1-deoxy D-mannose (**5**) (1.0 mM)/
LecB (550 μM) with 1-deoxy L-fucose
(**3**) (3 mM)

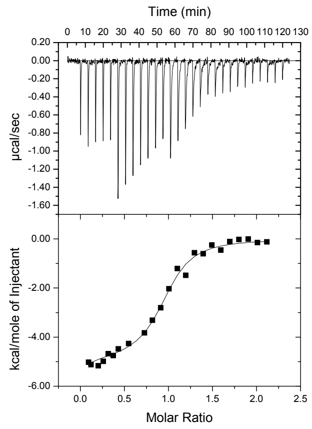

1-deoxy D-mannose (**5**) (4.0 mM)/
LecB (1.12 mM) with 1-deoxy L-
fucose (**3**) (10 mM)

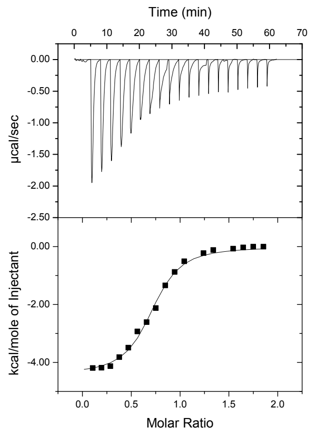

deoxyhybrid **(6)** (3.5 mM)
with LecB (387 μM)

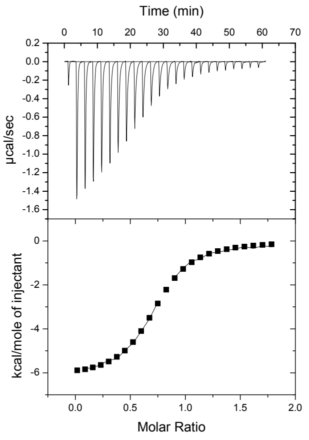

deoxyhybrid **(6)** (3.0 mM)
with LecB (405 μM)

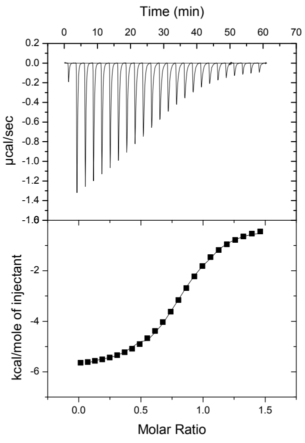

deoxyhybrid **(6)** (3.25 mM)
with LecB (396 μM)

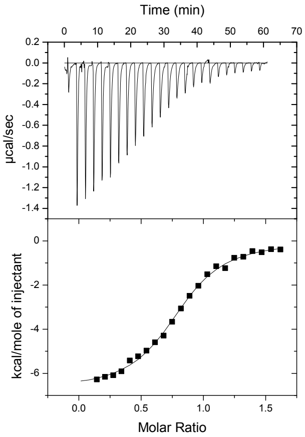

deoxyhybrid **(6)** (3.0 mM)
with LecB (307 μM)

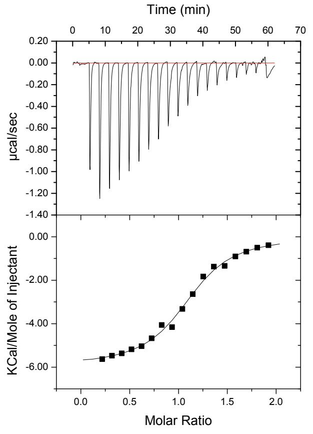

1-deoxy D-arabinose (**7**) (1.5 mM)
with LecB (116 μM)

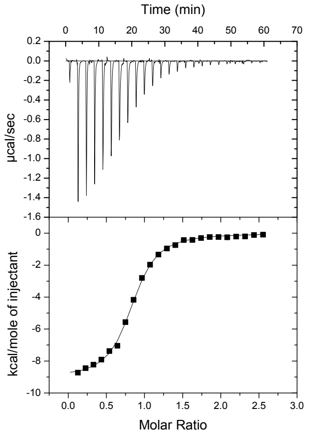

1-deoxy D-arabinose (**7**) (1.0 mM)
with LecB (121 μM)

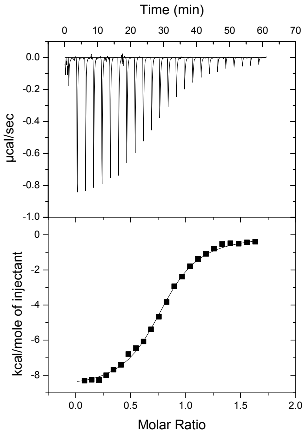

1-deoxy D-arabinose (**7**) (1.25 mM)
with LecB (132 μM)

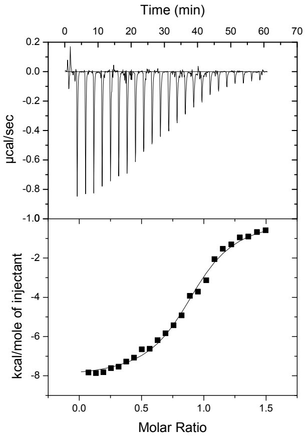

1-desoxy D-arabinose (**7**) (3.0 mM)
with LecB (307 μM)

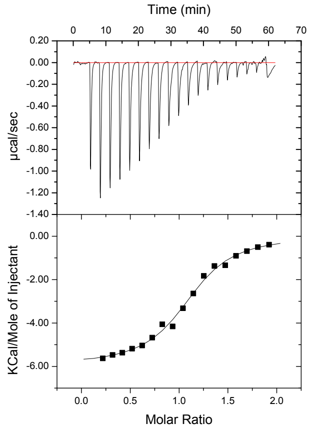

Experimental ITC data on the same systems may vary between different laboratories. For validation of the literature ITC data and to guarantee the validity of our comparisons between our data and data from the lab of Anne Imberty (Grenoble, France), we performed a titration of **1** with LecB. The data obtained are in very good agreement with the literature data by Sabin *et al*.[1]

Titration of LecB (56.1 μM) with **1** (700 μM):

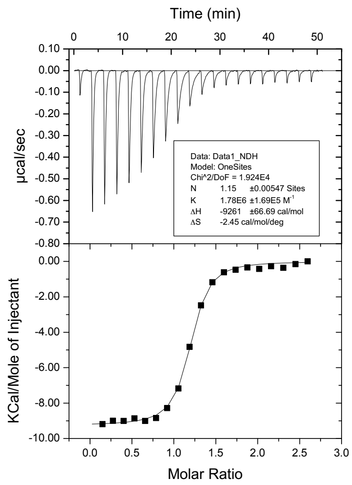

Thermodynamic parameters obtained:

ΔG (kJ/mol): -35.67, ΔH (kJ/mol): -38.73, TΔS (kJ*K/mol): -3.05, N: 1.15

Molecular Modeling

Flexibility of the Ligands in 15 ns MD Simulations of LecB Complexes:

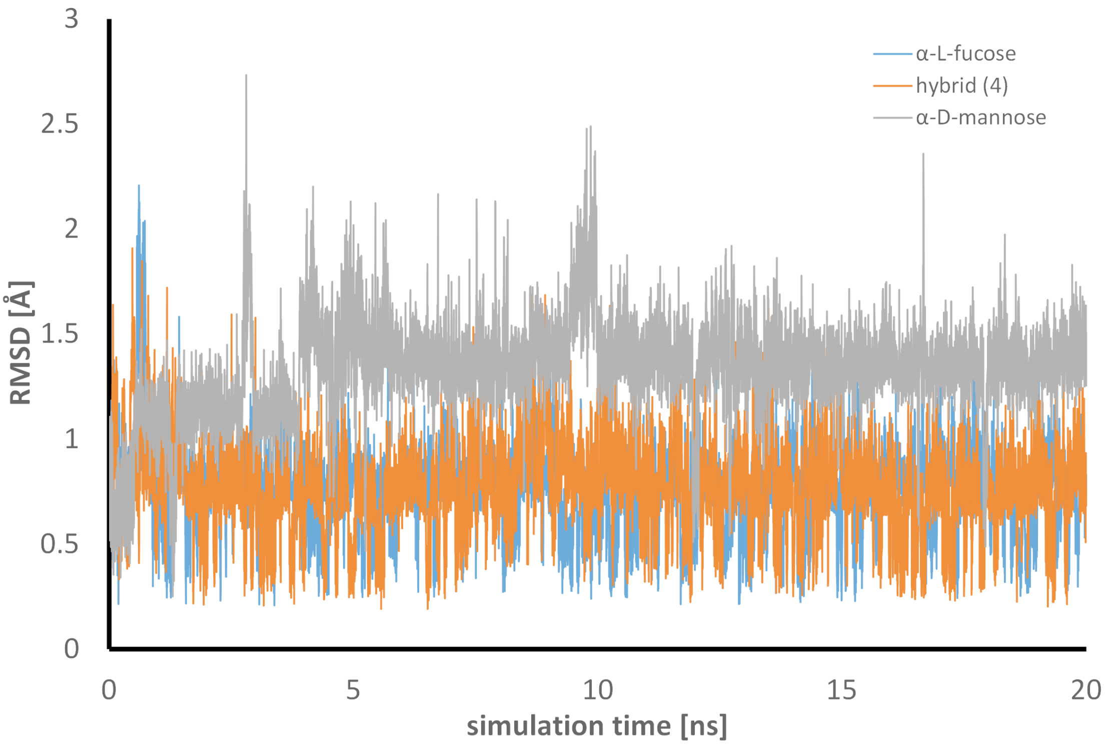

Figure S1: Time series of the root mean square deviation (RMSD) of all ligand atoms in the simulation of three complexes of LecB. Before the RMSD calculations the snapshots were aligned based on the binding site residues.

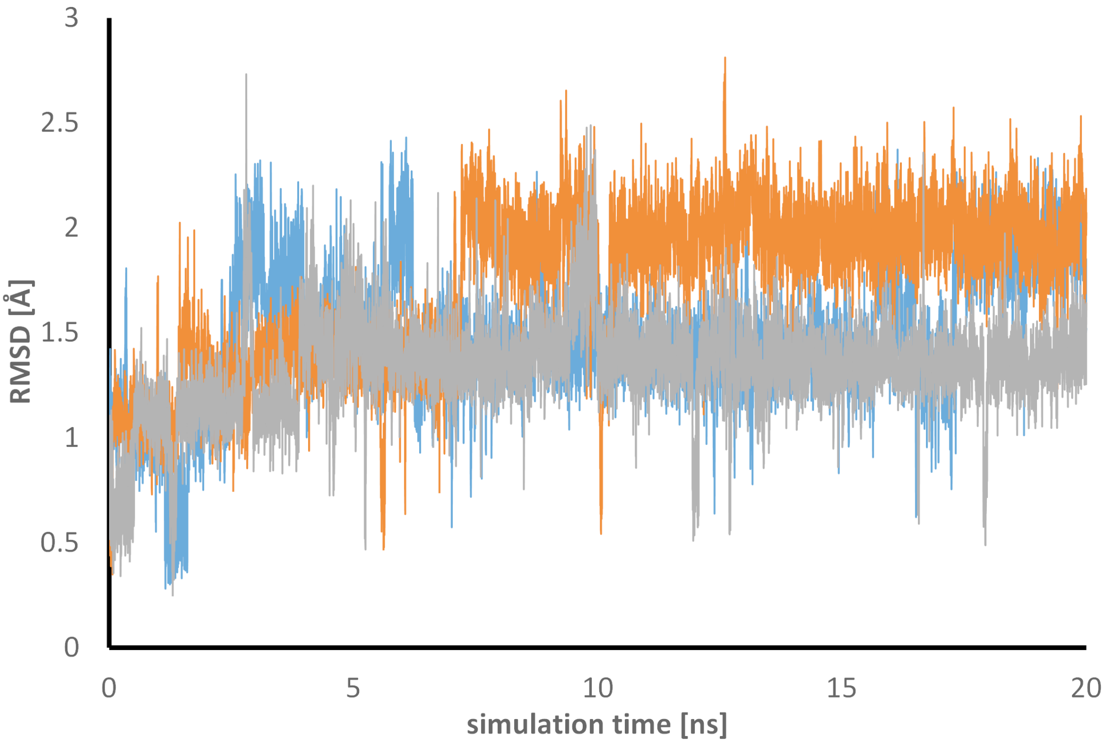

Figure S2: Time series of the root mean square deviation (RMSD) of all ligand atoms in the three independent simulation of three complexes of LecB with α-D-mannose. Before the RMSD calculations the snapshots were aligned based on the binding site residues.

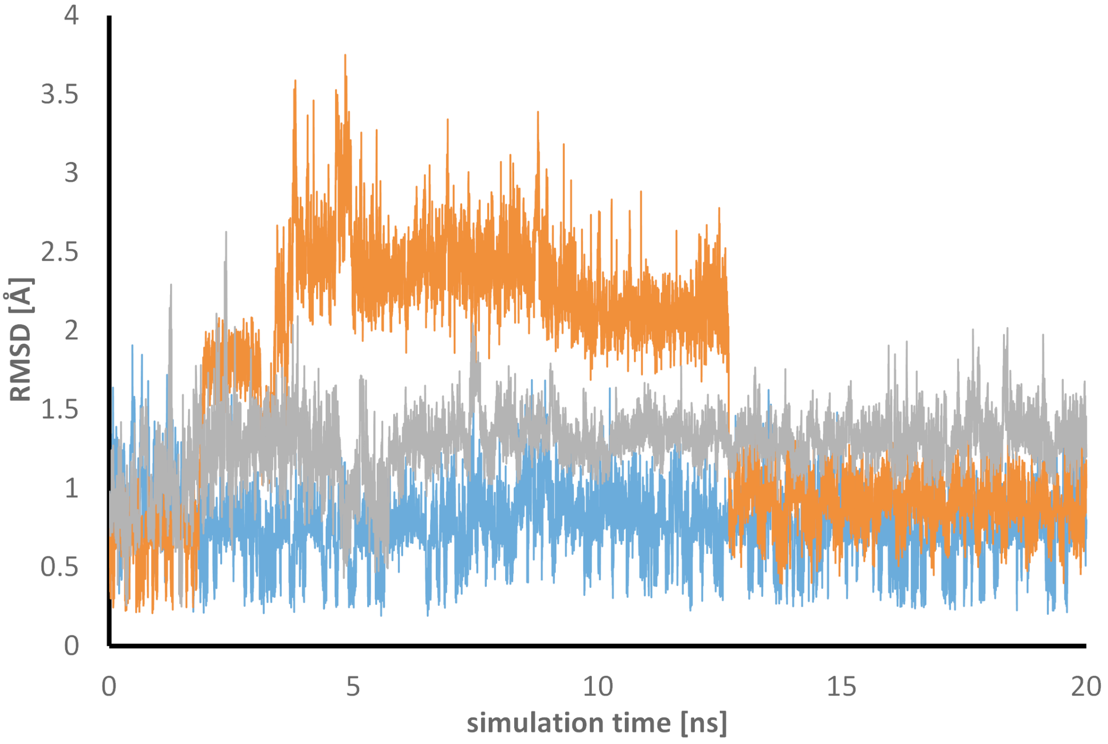

Figure S3: Time series of the root mean square deviation (RMSD) of all ligand atoms in the three independent simulation of three complexes of LecB with hybrid **4**. Before the RMSD calculations the snapshots were aligned based on the binding site residues.

Hydrogen Bond Between Asp96 and Ser22 in 15 ns Long MD Simulations of LecB Complexes:

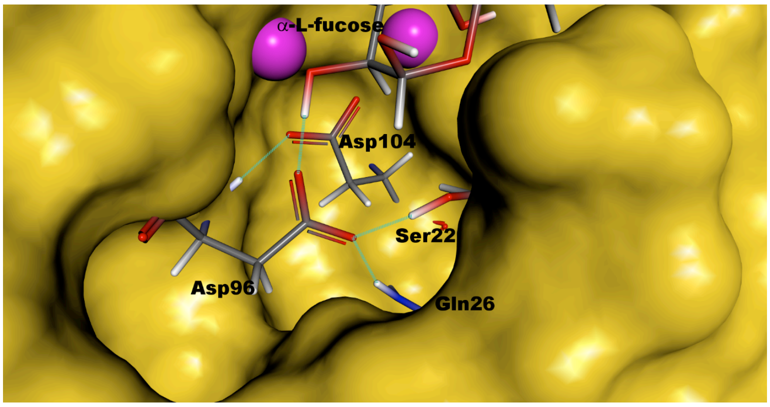

Figure S4: Full hydrogen-bond network of Asp96 with Ser22, Gln26, Asp104, and α-L-fucose. The calcium ions are shown as purple spheres.

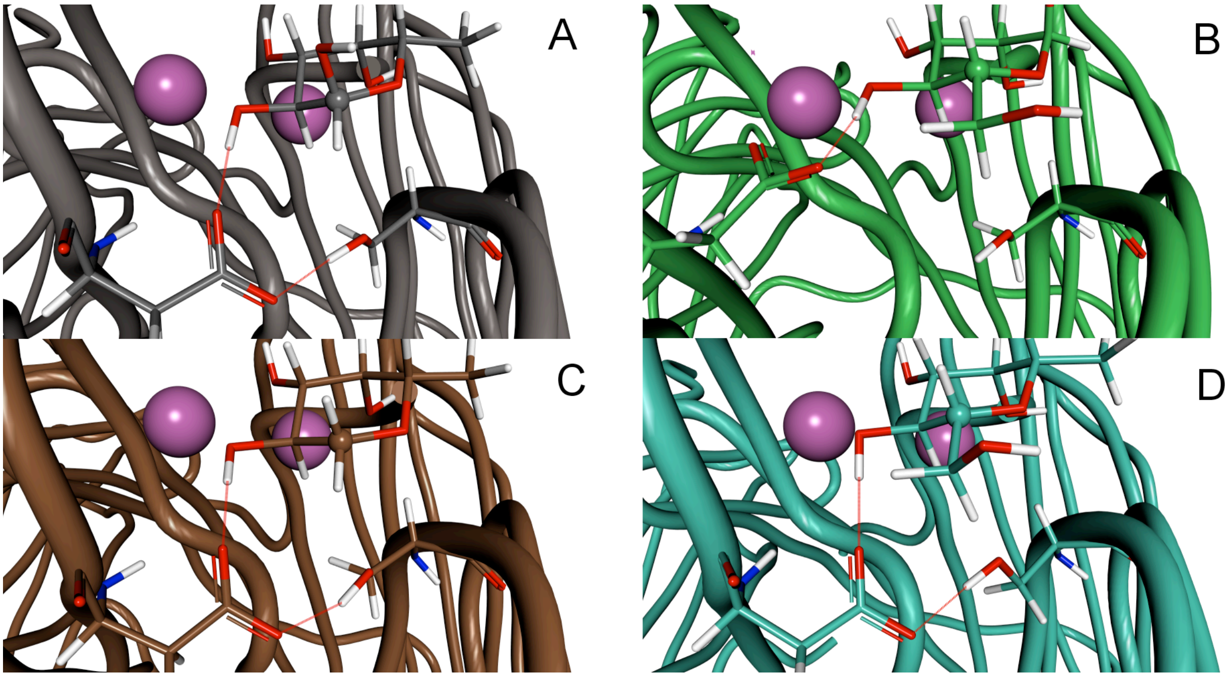

Figure S5: Orientation of Asp96 in the representative structure of the simulations of four complexes of LecB: (A) α-L-fucose (closed), (B) α-D-mannose (open), (C) 1-deoxy L-fucose (**3**, closed), (D) hybrid **4** (closed); The hydrogen bonds between Asp96 and Ser22 as well as Asp96 and the ligand are indicated by red dashed lines. The calcium ions are shown as purple spheres.

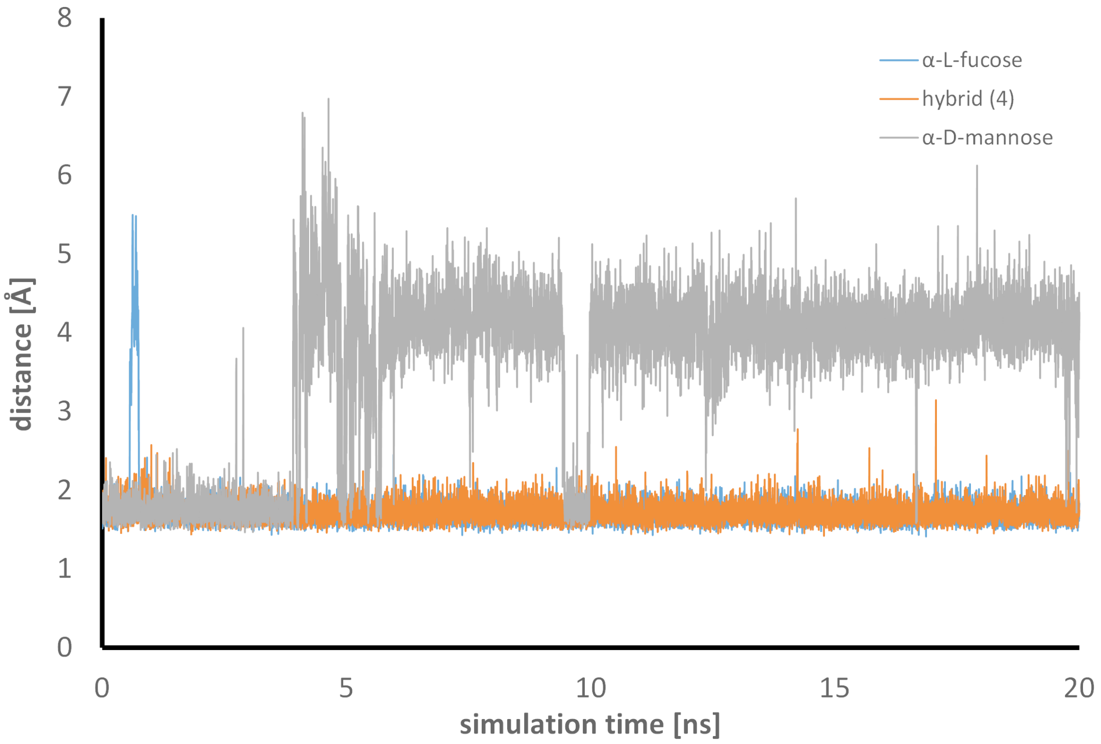

Figure S6: Time series of the shortest distance between HG of Ser22 and OD1/OD2 of Asp96 of simulations of three complex of LecB

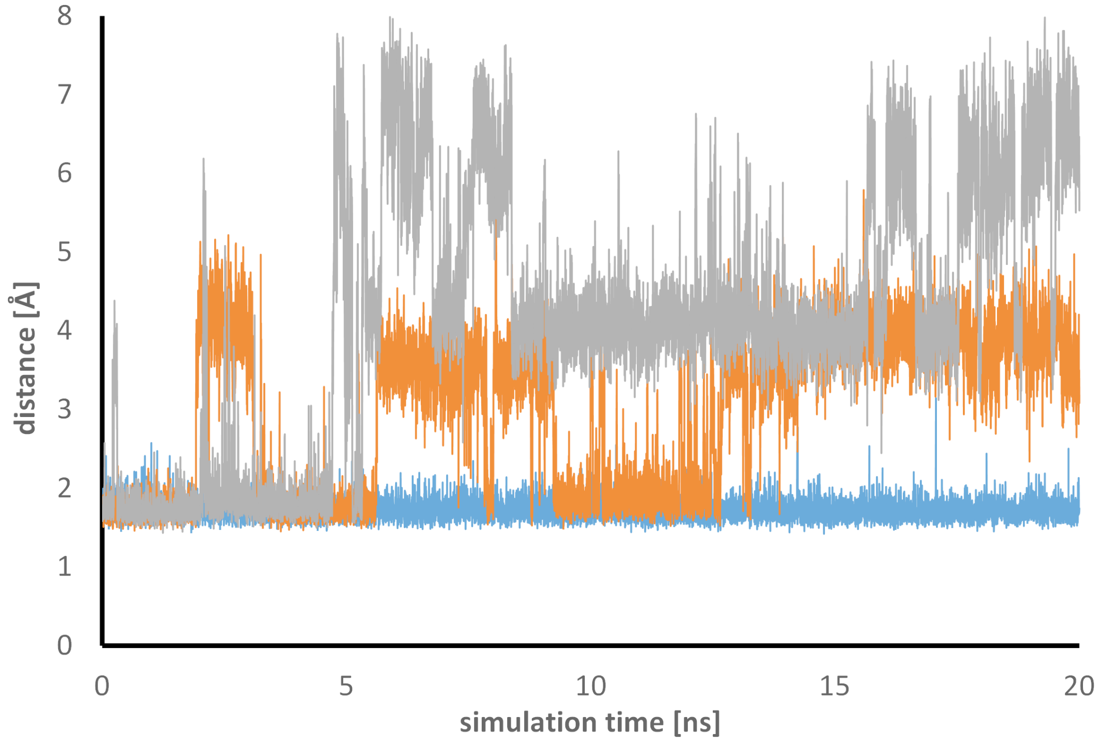

Figure S7: Time series of the shortest distance between HG of Ser22 and OD1/OD2 of Asp96 of three independent simulations of the complex of LecB with hybrid 4

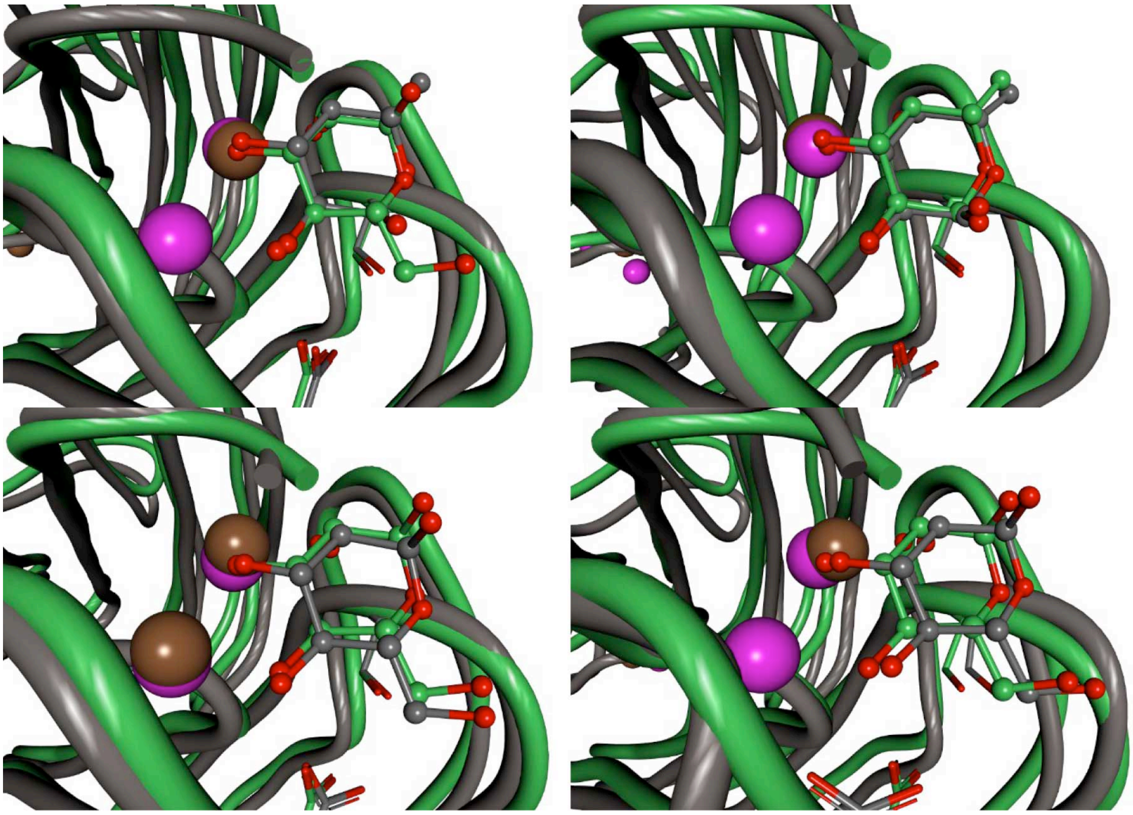

Figure S8: Overlays of experimental structures and representative structures from the MD simulations: The first structure is shown as a green ribbon, with green carbon atoms and with Ca(II) ions in magenta. The second structure is color coded in grey with brown Ca(II) ions. Upper left: X-ray structure of the fucose (1oxc) and mannose (1our) complex; Upper right: X-ray (1oxc) and MD structure of the fucose complex; Lower left: X-ray structure (1our) and closed form of the mannose complex; Lower right: X-ray structure (1our) and open form of the mannose complex

Contributions of Individual Steps of TI Simulation for Transition from 1-Deoxy L-Fucose (3) to Hybrid 4:

Table S1: Relative binding free energy for the transition from 1-deoxy L-fucose (**3**) to hybrid **4** calculated using thermodynamic integration and its partitioning into the free individual steps. The values for two independent calculations of 5 ns length as well as the averages are given.

**Step**	**Starting Structure 1 [kJ/mol]**	**Starting Structure 2 [kJ/mol]**	**Average [kJ/mol]**
Destruction of partial charges of vanishing group	1.32	1.39	1.35
Mutation of vanishing group into appearing group	13.10	14.78	13.94
Adding partial charges to appearing group	-3.48	-6.35	-4.92
**Sum**	10.93	9.81	10.37

Supplementary References

[1] Sabin C, Mitchell EP, Pokorná M, Gautier C, Utille J, Wimmerová M, Imberty A (2006) Binding of different monosaccharides by lectin PA-IIL from Pseudomonas aeruginosa: thermodynamics data correlated with X-ray structures. FEBS Lett 580: 982-7.
